# An innovative probabilistic hesitant fuzzy set MCDM perspective for selecting flexible packaging bags after the prohibition on single-use plastics

**DOI:** 10.1038/s41598-023-37200-2

**Published:** 2023-06-23

**Authors:** Jeonghwan Jeon, Suvitha Krishnan, Thangaraj Manirathinam, Samayan Narayanamoorthy, Mohammad Nazir Ahmad, Massimiliano Ferrara, Ali Ahmadian

**Affiliations:** 1 Department of Industrial and Systems Engineering, Gyeongsang National University, Jinju-si, Gyeongsangnam-do South Korea; 2 Department of Mathematics, Bharathiar University, Coimbatore, 641 046 India; 3 Institute of Visual Informatics, Universiti Kebangsaan Malaysia (UKM), 43600 Bangi, Selangor Malaysia; 4 Department of Law, Economics and Human Sciences, Mediterranea University of Reggio Calabria, Reggio Calabria, Italy; 5 School of Engineering and Technology, College of Engineering and Aviation, Central Queensland University, Rockhampton, Australia; 6 Department of Computer Science and Mathematics, Lebanese American University, Beirut, Lebanon; 7 Department of Mathematics, Near East University, Nicosia, Mersin 10, TRNC Turkey

**Keywords:** Applied mathematics, Environmental impact

## Abstract

The probabilistic hesitant elements (PHFEs) are a beneficial augmentation to the hesitant fuzzy element (HFE), which is intended to give decision-makers more flexibility in expressing their biases while using hesitant fuzzy information. To extrapolate a more accurate interpretation of the decision documentation, it is sufficient to standardize the organization of the elements in PHFEs without introducing fictional elements. Several processes for unifying and arranging components in PHFEs have been proposed so far, but most of them result in various disadvantages that are critically explored in this paper. The primary objective of this research is to recommend a PHFE unification procedure that avoids the deficiencies of operational practices while maintaining the inherent properties of PHFE probabilities. The prevailing study advances the hypothesis of permutation on PHFEs by suggesting a new sort of PHFS division and subtraction compared with the existing unification procedure. Eventually, the proposed PHFE-unification process will be used in this study, an innovative PHFEs based on the Weighted Aggregated Sum Product Assessment Method–Analytic Hierarchy Process (WASPAS–AHP) perspective for selecting flexible packaging bags after the prohibition on single-use plastics. As a result, we have included the PHFEs-WASPAS in our selection of the most effective fuzzy environment for bio-plastic bags. The ranking results for the suggested PHFEs-MCDM techniques surpassed the existing AHP methods in the research study by providing the best solution. Our solutions offer the best bio-plastic bag alternative strategy for mitigating environmental impacts.

## Introduction

In the concept of PHFS, probability is connected to the concept of HFS^1^. The strategy of PHFS, which can have a lot of potential in dealing with MCDM procedures in recent years, where the criteria are recognised in terms of achieving a set of performance goals. For example, using the qualitative flexible multiple (QUALIFLEX) technique, we predetermined the Hausdorff distance measure for (PHFEs) by using it to analyse green providers^2^. They created various PHFE distance initiatives in order to improve an interactive methodology for PHFEs-MCDM with inexact weight information. The presented a number of PHFE operations using Frank t-norm & t-conorm, which they then applied to a MCGDM^3^. The weighted PHFS paradigm was defined, as well as two weighted hesitant fuzzy aggregation operators depending upon this Archimedean t-norm or even t-conorm^4^. Evaluation studies and research on the PHFE concept can be observed in^5–8^. The two methods of normalisation procedures that are being proposed for PHFEs on this are probabilistic-normalization and cardinal-normalization^9^. It’s important to remember that all of these normalising methods should have an arbitrary aspect with nil probability, such a normalisation strategy clearly misrepresents the decision maker’s original data set(s). It’s worth noting that the two consecutive normalisation operations are performed before the aggregation step, despite the fact that they’re not required^2,10–12^, are among the most significant contributions addressing various types of probabilistic unification process, which is one of the first to characterise the probability part of a binary PHFE operation in terms of a diversity of comparable probabilities^13^. Recent works have attempted to develop a PHFEs-probabilistic unification process method that addresses the inadequacies of the previously mentioned techniques by^9,14^. The PHFS-probabilistic unification process will then be used to research the most environmentally sustainable varieties of plastic bags for packaging. The following is a summary of the study’s main motivation, Environmental damage has been engulfing the planet. Plastic waste is, without a doubt, one of the most massive environmental problems in society^15^. Plastic is used in a wide range of applications, from everyday items like drinking bottles, and plastic shopping bags to far most advanced ones^16^. According to reports, one million single-use plastic cups for having a drink and five trillion plastic containers for purchasing are acquired every minute and year, according to reports. Plastic’s increase is due to its low cost, versatility in applications, and complexity^17^. Plastic’s negative effects could’ve been contained if it hadn’t been used in such uncontrolled ways, it is not a material that is inherently harmful. The circular economy, as well as a potential remedy based on the development of public policies targeted at enhancing waste management and clearer legal guidelines for substitutes single-use plastics^18^. Industrial pollution has become so severe that it has been recommended as the Anthropocentric era’s geological indicator. Interruptions in the primary producers, pesticides in reservoirs, blighting of land, and emissions into the atmosphere are just a few of the negative consequences, all of which contribute to the destruction of the world’s biodiversity. Furthermore, both industrialized nations face the problem of disposing of waste material, up to 91% of all Styrofoam has not been reprocessed since the 1950s^19^. Because of the complexity of the situation, global-solving combination optimization to mitigate it has been proposed Hughes^20^. However, one system is the (GPAP), which was established at the WEF sustainability impact conference in 2012, to evaluate and optimize alternatives, use the fuzzy WASPAS-MCDM model in terms of multiple dimensions of sustainability, if there consider six different packaging bags with an emphasis on biodegradable plastic bags. Bio-plastic bags have always been found to be the most preferable option in the MCDM analysis, preceded by paper bags and plastic product bags. According to the findings of this study, using bio-plastic beverages instead of polythene bags would have a major impact on the environment. The nomenclature used in this manuscript is displayed in Table 1.

**Table 1:** Nomenclature.

HFS	Hesitant Fuzzy Set
PHFEs	Probabilistic Hesitant Fuzzy Elements
MCDM	Multi Criteria Decision Making
MCGDM	Multi Criteria Group Decision Making
GPAP	Global Plastic Action Partnership
PLA	polylactic acid
WASPAS	Weighted Aggregated Sum Product Assessment
ARAS	Additive Ratio Assessment
COPRAS	Complex Proportional Assessment
CODAS	Combinative Distance-based Assessment
AHP	Analytic Hierarchy Process
MOORA	Multi-Objective Optimization Ratio Analysis
PROMETHEE	Preference Ranking Organization Methods for Enrichment Evaluations
TOPSIS	Technique for Order Preference by Similarity to Ideal Solution

The remainder of this study is organized as follows: The literature review will be discussed in Section 2. The fundamental concepts are explained in Section 3. The proposed PHFEs-probabilistic unification process is in Section 4. The literature review would be followed by the methodology section in Section 5. The actual case study is presented in Section 6. The results and discussion, along with the conclusion, would be outlined in Section 7. Result validation would be discussed in Section 8. Conclusions and future work are all included in Section 9.

## Literature review

An MCDM frequently involves unpredictability, which could be explained by utilising fuzzy theory^21^. Among the most appropriate strategies for solving such problems is to use MCDM methods. Several researchers have described many techniques so far, such as, (COPRAS)^22^, (ELECTRE)^23^, (AHP)^24^, (PROMETHEE)^25^, (ANP)^26^, (SAM)^27^, (TOPSIS)^28^, (MULTIMOORA)^29^, (CODAS)^30^, (WASPAS)^31–33^. The HFS is a fuzzy set extension in which the psychological behavior of decision making is taken into account when determining membership was the one who first mentioned hesitant fuzzy set. In order to find practical solutions, researchers have recently invented and made decisions that are confusing and uncertain, solved an MCDM method in a fuzzy covering estimator space using the TOPSIS method and contributed to fuzzy set theory by presenting several approaches that make use of distinct fuzzy rough set models^34^. An Investigated the VIKOR technique for assessing hospital service quality in a fuzzy environment using linguistic factors parameterized by triangular fuzzy integers^14^. The MOORA approach is the most basic method for selecting the best option in a multi-objective optimization strategy^35^, proposed a workable solution for the linear case of a fuzzy decision matrix. The proposed various types of ranking methods for hesitant fuzzy evaluate for MADM and then invented the HFS and its features^12,36^. This issue is effectively resolved by the hesitant fuzzy set (HFS) put forth by^37^, which enables experts to accept a range of potential values as membership degrees. Following that, other researchers both domestically and internationally made significant advancements in HFS theory^6^. Multiple membership degrees are permitted in an element by HFS, but these membership degrees all start out with the same weight. However, different membership degrees actually equate to varying levels of relevance in the majority of cases because of expert choice and quantity. The probabilistic hesitant fuzzy set (PHFS)^13^, which adds matching probability information for each membership degree and so reflects varied importance, is developed to overcome this problem. PHFS, which is based on the fuzzy set and its extended forms, is a useful and efficient instrument that can increase the objectivity and legitimacy of the outcome. The PHFS theory has been expanded in recent years by scholars^9^. It is important to note that in these investigations, the probability that all membership degrees belong to the same probabilistic hesitant fuzzy element (PHFE) must add up to one. In truth, this criteria is frequently unsatisfiable in many real-world decision-making issues. As a result, Zhang et al. improved the concept of PHFS and decreased the requirements for probabilistic information. The uniqueness of this study derives from the fact that there is little analytical research focused on bio-plastic, a substitute for plastic bags. The purpose of fuzziness VIKOR is to discover a desirable compromise for decision-makers among different approaches based on many developed the term “fuzzy” to describe data that contains unpredictability^38^. The two criteria dealing with the discharge of pollutants into the atmosphere and hydrocarbons into the vacuum of space are CO2 and carbon pollution, these gases play a significant role in global warming^39^. All pollution from the recycling process until just before the component is redeployed to the purchasers was regarded as part of the GHG set of criteria. The term “global climate potential” refers to a method of contrasting the ecological effects of various carbon dioxide emissions^40^. The scope of traces left behind by a bag after its destiny has faded away is factored into the flexibility of recycling, bio-compatibility, and biodegradability of materials. According to studies, the density of moving furniture is extremely important, as lighter bags are more beneficial for customers. Plastic bags are prevalent for a variety of reasons, one of which is their breathable nature^41^. The findings of this study could also support other nations in making programmes to address the problems caused by plastic waste. Now that we have given it some thought, the proposed WASPAS technique offers high efficiency and effectiveness in decision-making. Its key benefits include computational simplicity, consistency of outcomes, and great resistance to alternate rank reversal. The fundamental benefit of the WASPAS approach, according to Hashemkhani Zolfani et al. is its high level of reliability. In everyday contradictory circumstances, the integration of rough statistics and the AHP-WASPAS technique, with the benefits of both concepts, provides crucial decision-making support^30^.

### Objective and motivation

In this study, decision-makers were given more fluidity in expressing their priorities, and then the probabilistic-unified type modified them with the normalised absorption of related probabilities. The fundamental goal of this research was to explore the implications of hesitantly relevant data in the practical decision-making process. In addition, the development and use of reasonably priced biodegradable plastic bags can save prospective customers and exporters money while also reducing environmental impact. Bio-plastic bags, which are made from non-fossil materials, can be a suitable alternative to plastic bags. Their use helps to address issues that are commonly associated with petrochemical plastics, such as the global energy crisis, volatile oil prices, and negative effects on global temperature. The problem-solving section of MCDM is responsible for the selection of projects based on a variety of criteria. Determine all aspects of these alternatives, from advantages to disadvantages, using all criteria. As a result, the primary motivation for this research paper is to identify the most selected flexible packaging bag after the prohibition of single-use plastics using an MCDM system with a probabilistic hesitant set.

### Contribution of the research work

This work’s main contribution is the capture of a novel unified procedure that has been developed for PHFEs. After that, in the MCDM technique, selecting the best replacement bags for packaging instead of plastic bags necessitates the use of a special mathematical set theory. We chose the hesitant fuzzy set developed by Torra^37^ and the unification method for PHFS as our contribution to this publication. In the weighting section, we used a PHFE in the AHP weight-finding strategy, which is an essential feature of the MCDM method. As a result, we have included the PHFE in our selection of the most ecologically responsible form of plastic bag in a hesitant fuzzy environment. The well-known “MCDM-WASPAS” technique was used in selecting the most ecologically responsible plastic bag types. The ranking results for the suggested WASPAS-MCDM techniques surpassed the existing PHFS-MCDM methods in the research study by providing the best solution. According to the comparative study, the four approaches (CODAS, COPRAS, ARAS, and MOORA) to the PHFS environment have perfect rankings. The sensitivity analysis examines the ranking order when subjective weights’ importance is changed in two cases. Our solutions offer the best bio-plastic bag alternative strategy for mitigating environmental impacts.

## Preliminaries

we recall those basic definition which are used frequently and then discuss the arithmetic operations involved in dealing with PHFEs.

### Definition 1

^12^ Let $\Re$ be the universal set, defined by the hesitant fuzzy set (HFS) on $\Re$ in finite subset of [0,1].

$$\begin{aligned} G=\{\langle {x},g(x)\rangle / x \in \Re \} \end{aligned}$$

where $g(x) \in$ [0,1], which denotes the element’s possible membership degree $x \in$ $\Re$ to the set G.

In the HFS, G can be represented in terms of

1 $$\begin{aligned} G =\left\{ \left\langle x,\bigcup _{\textit{g} \in g(x)}\{\textit{g} \}\right\rangle / x\in \Re \right\} \end{aligned}$$

Let $g=\bigcup _{{\textit{g} } \in {g}}\{{\textit{g} } \}, g_1=\bigcup _{{\textit{g} _1} \in {g_1}}\{{\textit{g} _1} \}$ and $g_2=\bigcup _{{\textit{g} _2} \in {g_2}}\{{\textit{g} _2} \}$ are implied to be three HFEs, then the following operations on them are identified,

$$\begin{aligned}&g^c=\bigcup _{\textit{g} \in h}\{1-\textit{g} \}\\&g_1\cup g_2=\bigcup _{\textit{g} _1\in g_1,\textit{g} _2\in g_2} \max \{\textit{g} _1,\textit{g} _2\};\qquad g_1\cap g_2=\bigcup _{\textit{g} _1 \in g_1,\textit{g} _2\in g_2}\min \{\textit{g} _1,\textit{g} _2\}\\&g^\kappa =\bigcup _{\textit{g} \in g}\{\textit{g}^\kappa \};\qquad \kappa g =\bigcup _{\textit{g} \in g}\{1-(1-\textit{g} )^\kappa \} \quad \kappa >0\\&g_1\oplus g_2=\bigcup _{\textit{g} _1\in g_1,\textit{g} _2\in g_2} \{\textit{g} _1+\textit{g} _2-\textit{g}_1\textit{g} _2\};\qquad g_1\otimes g_2 =\bigcup _{\textit{g} _1\in g_1,\textit{g} _2\in h_2}\{\textit{g} _1\textit{g} _2\} \end{aligned}$$

### Definition 2

^42^ It was decided to use the probabilistic hesitant fuzzy set (PHFS), which shows that each potential value of HFE can be connected to an actual probability value. The PHFS on $\Re$ was defined as,

2 $$\begin{aligned} {}^\gamma {G}=\{\langle x,{}^\gamma {g}(x)\rangle :x\in \Re \}=\left\{ \left\langle x,\bigcup _{\langle {\textit{g} }(x) , \gamma (x)\rangle \in {}^\gamma {g}(x)}\{\langle {\textit{g} }(x) , \gamma (x)\rangle \}\right\rangle / x\in \Re \right\} \end{aligned}$$

A PHFEs, $^\gamma {g}(x)$ constitute both of membership degrees x $\in \Re$ being defined by ${\textit{g} }$(x) in addition to the probability of $\gamma$(x)$\in$ [0,1], such that $\sum _{^\gamma {g(x)}} \gamma (x)=1$ for any x $\in \Re .$

If $$^\gamma {g}=\bigcup _{\langle {\textit{g} } , \gamma \rangle \in {}^\gamma {g}}\{\langle {\textit{g} } , \gamma \rangle \}, {}^\gamma g_1=\bigcup _{\langle {\textit{g}_1} , \gamma _1\rangle \in {}^\gamma {g_1}}\{\langle {\textit{g} _1} , \gamma _1\rangle \}$$ and $${}^\gamma g_2=\bigcup _{\langle {\textit{g} _2} , \gamma _2\rangle \in {}^\gamma {g_2}}\{\langle {\textit{g} _2} , \gamma _2\rangle \}$$

Consider the three PHFEs,the presenting some operations,

$$\begin{aligned} {}^\gamma g^\kappa =&{} \bigcup _{\langle {\textit{g} } , \gamma \rangle \in ^\gamma {g}}\{\langle {\textit{g} }^\kappa , \gamma \rangle \}\\ \kappa ^\gamma g =&\bigcup _{\langle {\textit{g} } , \gamma \rangle \in ^\gamma {g}}\{\langle 1-(1-{\textit{g} })^\kappa , \gamma \rangle \}\\ ^\gamma {g}_1\oplus ^\gamma g_2=&\bigcup _{\langle {\textit{g} _1} , \gamma _1\rangle \in ^\gamma {g_1},\langle {\textit{g}_2} , \gamma _2\rangle \in ^\gamma {g_2}}\{\langle 1-(1-{\textit{g} _1})(1-{\textit{g} _2}) , \gamma _1 \gamma _2\rangle \}\\ ^\gamma {g}_1\otimes {}^\gamma {g}_2=&\bigcup _{\langle {\textit{g} _1} , \gamma _1\rangle \in ^\gamma {g_1},\langle {\textit{g} _2} , \gamma _2\rangle \in ^\gamma {g_2}}\{\langle {\textit{g}_1}{\textit{g} _2} , \gamma _1 \gamma _2\rangle \} \end{aligned}$$

### Definition 3

^43^ Let $^\gamma g(x)=\left\{ \gamma _{i}\left( g_{i}\right) / i=1,2, \ldots , \# g \right\}$ a PHFEs,the score (mean value) function is defined by:

$$\begin{aligned} S\left( ^\gamma g(x)\right) =\sum _{i=1}^{\# g} \gamma _{i} g_{i}. \end{aligned}$$

where $\gamma _i$ is the probability of possible value $^\gamma g(x)$ exhilarating $\sum _{i=1}^{\# g} \gamma _{i} =1$ ,# g is the number of different PHFEs in $^\gamma g(x)$.

## The unification process of PHFEs

In this attribute of importance, we will develop a unique optimization technique for performing the probabilistic unification process. Using this approach, the initial partition of each PHFE probability performs quite well, resulting in all associated PHFEs having the same probability aspects while their correlating HFE part remains constant.

Suppose that ${}^\gamma g_1=\bigcup _{\langle {\textit{g} _1} ,\gamma _1\rangle \in {}^\gamma {g_1}}\{\langle {\textit{g} _1},\gamma _1\rangle \}, {}^\gamma g_2=\bigcup _{\langle {\textit{g} _2} ,\gamma _2\rangle \in {}^\gamma {g_2}}\{\langle {\textit{g} _2} ,\gamma _2\rangle \},\ldots ,$ and ${}^\gamma g_m=\bigcup _{\langle {\textit{g} _m} ,\gamma _m\rangle \in {}^\gamma {g_m}}\{\langle {\textit{g} _m},\gamma _m\rangle \}$ are m arbitrary PHFEs whose probabilities set is

$\{\gamma ^{1}_1,\gamma ^{2}_1,\ldots ,\gamma ^{p_1}_1\}, \{\gamma ^{1}_2,\gamma ^{2}_2,\ldots ,\gamma ^{p_2}_2\},\ldots ,$ and $\{\gamma ^{1}_m,\gamma ^{2}_m,\ldots ,\gamma ^{p_m}_m\},$ respectively. The following is a description of the fresh probability-based methodology:

**Algorithm 1.**

(A procedure for unifying probabilities - PHFEs.)

*The first step* Insert

$$\begin{aligned} \left\{ \begin{array}{l} \{\gamma ^{1}_1,\gamma ^{2}_1,\ldots ,\gamma ^{p_1}_1\}; \\ \{\gamma ^{1}_2,\gamma ^{2}_2,\ldots ,\gamma ^{p_2}_2\};\\ \vdots \\ \{\gamma ^{1}_m,\gamma ^{2}_m,\ldots ,\gamma ^{p_m}_m\}. \end{array} \right. \end{aligned}$$

*Stage 1* Let i=1

$$\begin{aligned} \gamma ^i_{*}=\min \{\gamma ^{i}_1,\gamma ^{i}_2,\ldots ,\gamma ^{i}_m\}. \end{aligned}$$

*Stage 2*

$$\begin{aligned} \left\{ \begin{array}{l} \gamma ^{i}_1 =\max \{\gamma ^{i}_1-\gamma ^i_{*},0\}; \\ \gamma ^{i}_2 =\max \{\gamma ^{i}_2-\gamma ^i_{*},0\};\\ \vdots \\ \gamma ^{i}_m =\max \{\gamma ^{i}_m-\gamma ^i_{*},0\}. \end{array} \right. \end{aligned}$$

If $\gamma ^{i}_1=\gamma ^{i}_2=\cdots =\gamma ^{i}_m=0,$ then finish & let us take $\gamma _{*}=\{\gamma ^{1}_*,\gamma ^{2}_*,\ldots ,\gamma ^{p_*}_*\}$

where $p_*\ge \max \{p_1,p_2,\ldots ,p_m\},$ otherwise goto Stage 3.

*Stage 3* Define

$$\begin{aligned} \gamma ^{i}_j = \left\{ \begin{array}{ll} \gamma ^{i+1}_j &{}{} if \quad \gamma ^{i}_j=0 \\ \gamma ^{i}_j &{}{} if \quad \gamma ^{i}_j\not =0 \\ \end{array} \right. \end{aligned}$$

*Stage 4* We have i=i+1 then goto stage 1.

Additionally, we wish to emphasise that the probabilistic unification process indicated above is degraded into the steps listed below for clarity’s purpose:

*Case 1* Obtaining the probabilistic unification process $\{\gamma ^{1}_*,\gamma ^{2}_*,\ldots ,\gamma ^{l_*}_*\}$ by using Algorithm 1.

*Case 2* Re-arranging the PHFEs ${}^\gamma g_1,{}^\gamma g_2,\ldots ,$ and ${}^\gamma g_m$ into a unified term of ${}^\gamma {{\dot{g}}}_1,{}^\gamma {{\dot{g}}}_2,\ldots ,$ and ${}^\gamma {{\dot{g}}}_m,$ respectively.

The UPS is $\gamma _{*}=\{\gamma ^{1}_*,\gamma ^{2}_*,\ldots ,\gamma ^{l_*}_*\}$ the PHFEs can be preserved in the following way:

Suppose that the set of PHFEs ${}^\gamma g_1=\bigcup _{\langle {\textit{g} _1} ,\gamma _1\rangle \in {}^\gamma {g_1}}\{\langle {\textit{g} _1},\gamma _1\rangle \}, {}^\gamma g_2=\bigcup _{\langle {\textit{g} _2} , \gamma _2\rangle \in {}^\gamma {g_2}}\{\langle {\textit{g} _2} ,\gamma _2\rangle \},\ldots ,$ and ${}^\gamma g_m=\bigcup _{\langle {\textit{g} _m} ,\gamma _m\rangle \in {}^\gamma {g_m}}\{\langle {\textit{g} _m},\gamma _m\rangle \}$ as below:

$$\begin{aligned} \left\{ \begin{array}{l} \{\gamma ^{1}_1,\gamma ^{2}_1,\ldots ,\gamma ^{p_1}_1\}; \\ \{\gamma ^{1}_2,\gamma ^{2}_2,\ldots ,\gamma ^{p_2}_2\};\\ \vdots \\ \{\gamma ^{1}_m,\gamma ^{2}_m,\ldots ,\gamma ^{p_m}_m\}. \end{array} \right. \end{aligned}$$

We also consider the UPS in order to keep the debate simple. This is the case,

$$\begin{aligned}&\gamma ^{1}_1=\sum _{s=1}^{s_1}\gamma ^{s}_* ,\\&\gamma ^{2}_1=\sum _{s=s_1+1}^{s_2} \gamma ^{s}_*,\\&\vdots \\&\gamma ^{p_1}_1=\sum _{s=s_{p_1}+1}^{{p_1}} \gamma ^{s}_*, \end{aligned}$$

The HFE part of the preliminary PHFE is then sorted as follows:

$$\begin{aligned}&\langle {{\textit{g}^1_1}},\gamma ^{1}_*\rangle ,\ldots \langle {{\textit{g}^1_1}},\gamma ^{s_1}_*\rangle ,\\&\langle {{\textit{g}^2_1}},\gamma ^{s_1+1}_*\rangle ,\ldots \langle {{\textit{g}^2_1}},\gamma ^{s_2}_*\rangle ,\\&\vdots \\&\langle {{\textit{g}^{p_1}_1}},\gamma ^{s_{p_1}+1}_*\rangle ,\ldots \langle {{\textit{g}^{p_1}_1}},\gamma ^{s_{p^*}}_*\rangle . \end{aligned}$$

The unified structure of the PHFE ${}^\gamma g_1$ will be

3 $$\begin{aligned} {}^\gamma {{\dot{g}}}_1=&{} \{\langle {{{\dot{\textit{g} }}^1_1}},{{\dot{\gamma }}}^{1}_1\rangle ,\ldots \langle {{{\dot{\textit{g} }}^{s_1}_1}},{{\dot{\gamma }}}^{s_1}_1\rangle ,\nonumber \\ {}&\langle {{{\dot{\textit{g} }}^{s_1+1}_1}},{{\dot{\gamma }}}^{s_1+1}_1 \rangle ,\ldots \langle {{{\dot{\textit{g} }}^{s_2}_1}},{{\dot{\gamma }}}^{s_2}_1\rangle ,\nonumber \\ {}&\vdots \nonumber \\ {}&\langle {{{\dot{\textit{g} }}^{s_{p_1}+1}_1}},{{\dot{\gamma }}}^{s_{p_1} +1}_1\rangle ,\ldots \langle {{{\dot{\textit{g} }}^{p^*}_1}},{\dot{\gamma }}^{p^*}_1\rangle \} \end{aligned}$$

### Corollary 1

Let ${}^\gamma {{\dot{g}}}_1=\bigcup _{\langle {{\dot{\textit{g} }}_1} ,{{\dot{\gamma }}}_1\rangle \in {}^\gamma {g_1}}\{\langle {{\dot{\textit{g} }}_1},{{\dot{\gamma }}}_1\rangle \}$ and ${}^\gamma {{\dot{g}}}_2=\bigcup _{\langle {{\dot{\textit{g} }}_2} ,{\dot{\gamma }}_2\rangle \in {}^\gamma {g_2}}\{\langle {{\dot{\textit{g} }}_2} ,{{\dot{\gamma }}}_2\rangle \}$ are two UPHFEs. The sets of probabilities that correspond to them are then compatible.

### Proof

The re-formatting of probabilities shows that the proof is correct. ${\gamma }_1$ and ${\gamma }_2$ in the morphology of ${\dot{\gamma }}_1={{\dot{\gamma }}}_2=\gamma _*.$ The proposed PHFE-probabilistic unification process. Suppose that

$$\begin{aligned} {}^\gamma g_1=&{} \{\langle {\textit{g}_1^1},\gamma ^1_1\rangle ,\langle {\textit{g}_1^2},\gamma _1^2\rangle ,\langle {\textit{g} _1^3},\gamma _1^3\rangle \},\\ {}^\gamma g_2=&{} \{\langle {\textit{g}^1_2},\gamma ^1_2\rangle ,\langle {\textit{g} _2^2},\gamma _2^2\rangle \}, \\ {}^\gamma g_3=&{} \{\langle {\textit{g}^1_3},\gamma ^1_3\rangle ,\langle {\textit{g} _3^2},\gamma _3^2\rangle ,\langle {\textit{g} _3^3},\gamma _3^3\rangle \} \end{aligned}$$

are three PHFEs chosen at random. Then, as described in the following, their unified methods can be deduced:

$$\begin{aligned} {}^\gamma {{\dot{g}}}_1=&{} \{\langle {\textit{g}^1_1},\gamma ^1_*\rangle ,\langle {\textit{g} _1^1},\gamma _*^2\rangle ,\langle {\textit{g} _1^2},\gamma _*^3\rangle ,\langle {\textit{g} _1^2},\gamma _*^4\rangle ,\langle {\textit{g} _1^3},\gamma _*^5\rangle ,\langle {\textit{g} _1^3},\gamma _*^6\rangle \},\\ {}^\gamma {{\dot{g}}}_2=&{} \{\langle {\textit{g}^1_2},\gamma ^1_*\rangle ,\langle {\textit{g}_2^1},\gamma _*^2\rangle ,\langle { \textit{g}_2^1},\gamma _*^3\rangle ,\langle {\textit{g} _2^2},\gamma _*^4\rangle ,\langle {\textit{g} _2^2},\gamma _*^5\rangle ,\langle {\textit{g} _2^2},\gamma _*^6\rangle \},\\ {}^\gamma {{\dot{g}}}_3=&{} \{\langle {\textit{g}^1_3},\gamma ^1_*\rangle ,\langle {\textit{g} _3^2},\gamma _*^2\rangle ,\langle {\textit{g} _3^2},\gamma _*^3\rangle ,\langle {\textit{g} _3^2},\gamma _*^4\rangle ,\langle {\textit{g} _3^2},\gamma _*^5\rangle ,\langle {\textit{g} _3^3},\gamma _*^6\rangle \}. \end{aligned}$$

As previously stated, the two phases that follow could easily yield these illustrated results:

*Case 1* The unification process set is $\{\gamma ^{1}_*,\gamma ^{2}_*,\ldots ,\gamma ^{6}_*\}$ is extracted by the use of unification process (Algorithm 1).

**Figure 1:**
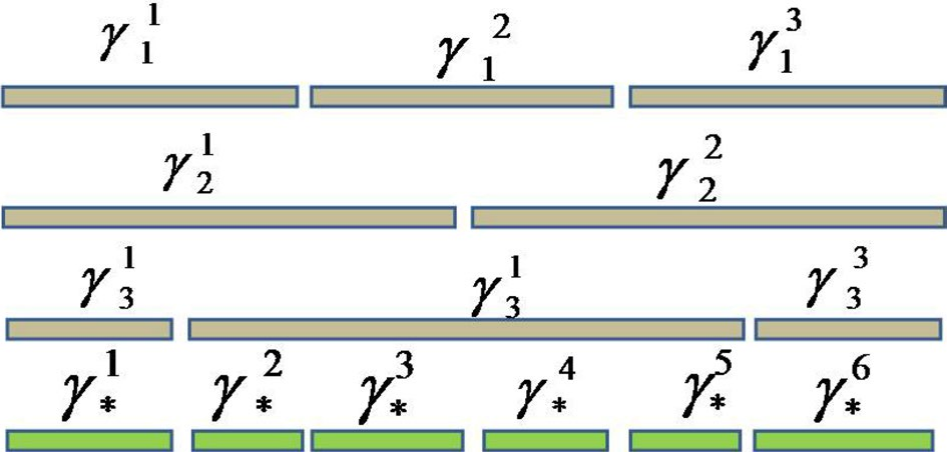
The first case in the unification process.

**Figure 2:**
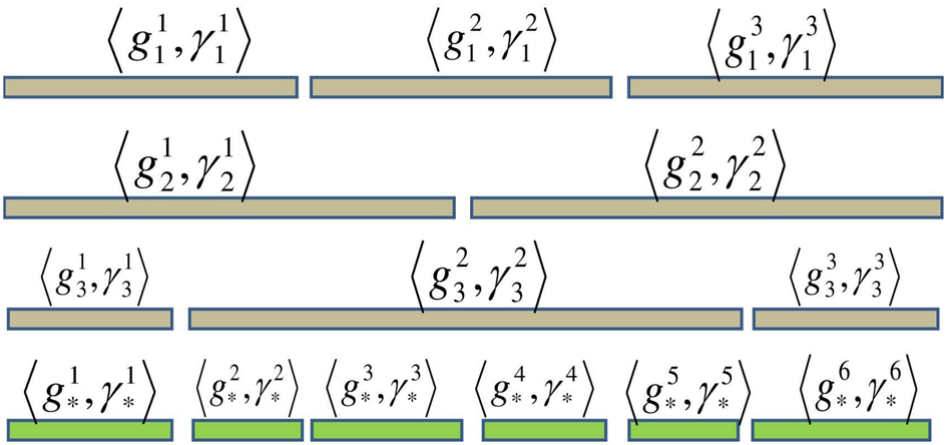
The second case in the unification process.

**Figure 3:**
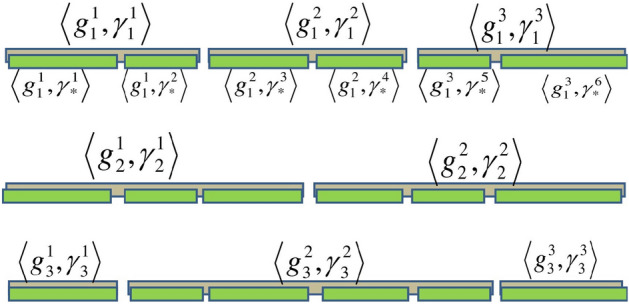
Embracing the first and second cases of the unification process.

*Case 2* So finally, Case 1 are used to explain how such PHFEs are re-formatted. ${}^\gamma g_1,{}^\gamma g_2$ and ${}^\gamma g_3$ to obtain the UPHFEs forms of ${}^\gamma {{\dot{g}}}_1,{}^\gamma {{\dot{g}}}_2$ and ${}^\gamma {{\dot{g}}}_3.$ Figures 1, 2 and 3, show the data drawn on how to perform cases 1 and 2.

### Operations based on the UPHFE

Let ${}^\gamma {{\dot{g}}}_1=\bigcup _{\langle {{\dot{\textit{g} }}_1} ,{{\dot{\gamma }}}_1\rangle \in {}^\gamma {g_1}}\{\langle {{\dot{\textit{g} }}_1},{{\dot{\gamma }}}_1\rangle \}$ and ${}^\gamma {{\dot{g}}}_2=\bigcup _{\langle {{\dot{\textit{g} }}_2} ,{\dot{\gamma }}_2\rangle \in {}^\gamma {g_2}}\{\langle {{\dot{\textit{g}}}_2} ,{{\dot{\gamma }}}_2\rangle \},$ are two UPHFEs. Then, the following some operations of PHFEs can be introduced.

*(i) Addition *

4 $$\begin{aligned} {}^\gamma {{\dot{g}}}_1\oplus {}^\gamma {{\dot{g}}}_2= {} \{\langle 1-(1-{{\dot{\textit{g} }}^1_1})(1-{{\dot{\textit{g} }}^1_2}) ,{{\gamma }_*^{1}}\rangle ,\ldots ,\langle 1-(1-{{\dot{\textit{g} }}^{p^*}_1})(1-{{\dot{\textit{g} }}^{p^*}_2}) ,{{\gamma }_*^{p^*}}\rangle \} \end{aligned}$$

*(ii) Multiplication*

5 $$\begin{aligned} {}^\gamma {{\dot{g}}}_1\oplus {}^\gamma {{\dot{g}}}_2= {} \{\langle {{\dot{\textit{g} }}^1_1}{{\dot{\textit{g} }}^1_2} ,{{\gamma }_*^{1}}\rangle ,\ldots ,\langle {{\dot{\textit{g} }}^{p^*}_1}{{\dot{\textit{g} }}^{p^*}_2} ,{{\gamma }_*^{p^*}}\rangle \} \end{aligned}$$

*(iii) Division*

6 $$\begin{aligned} {}^\gamma {{\dot{g}}}_1\oslash {}^\gamma {{\dot{g}}}_2=&{} \left\{ \left\langle \min \left\{ 1,\frac{{{\dot{\textit{g} }}^{1}_1}}{{{\dot{\textit{g} }}^{1}_2}}\right\} ,\gamma ^{1}_*\right\rangle ,\ldots \left\langle \min \left\{ 1,\frac{{{\dot{\textit{g} }}^{p^*}_1}}{{{\dot{\textit{g}}}^{l^*}_2}}\right\} ,\gamma ^{p^*}_*\right\rangle \right\} \end{aligned}$$

*(iv) Subtraction*

7 $$\begin{aligned} {}^\gamma {{\dot{g}}}_1\ominus {}^\gamma {{\dot{g}}}_2=&{} \left\{ \left\langle \max \left\{ 0,\frac{{{\dot{\textit{g} }}^{1}_1}-{{\dot{\textit{g} }}^{1}_2}}{1-{{\dot{\textit{g} }}^{1}_2}} \right\} ,\gamma ^{1}_*\right\rangle ,\ldots \left\langle \max \left\{ 0,\frac{{{\dot{\textit{g} }}^{p^*}_1}-{{\dot{\textit{g} }}^{p^*}_2}}{1-{{\dot{\textit{g}}}^{p^*}_2}} \right\} ,\gamma ^{p^*}_*\right\rangle \right\} \end{aligned}$$

*Properties 1.*

Let $${}^\gamma {{\dot{g}}}_1=\bigcup _{\langle {{\dot{\textit{g} }}_1} ,{{\dot{\gamma }}}_1\rangle \in {}^\gamma {g_1}}\{\langle {{\dot{\textit{g} }}_1},{{\dot{\gamma }}}_1\rangle \}, {}^\gamma {{\dot{g}}}_2=\bigcup _{\langle {{\dot{\textit{g} }}_2} ,{{\dot{\gamma }}}_2\rangle \in {}^\gamma {g_2}}\{\langle {{\dot{\textit{g} }}_2} ,{{\dot{\gamma }}}_2\rangle \},$$ and

${}^\gamma {{\dot{g}}}_3=\bigcup _{\langle {{\dot{\textit{g} }}_3} ,{{\dot{\gamma }}}_3\rangle \in {}^\gamma {g_3}}\{\langle {{\dot{\textit{g} }}_3},{{\dot{\gamma }}}_3\rangle \}$ are three UPHFEs. Then, we consider that

$$\begin{aligned}&({}^\gamma {{\dot{g}}}_1\oplus {}^\gamma {{\dot{g}}}_2)\ominus ({}^\gamma {{\dot{g}}}_2\oplus {}^\gamma {{\dot{g}}}_3)=({}^\gamma {{\dot{g}}}_1\ominus {}^\gamma {{\dot{g}}}_3)\\&({}^\gamma {{\dot{g}}}_1\otimes {}^\gamma {{\dot{g}}}_2)\oslash ({}^\gamma {{\dot{g}}}_2\otimes {}^\gamma {{\dot{g}}}_3)=({}^\gamma {{\dot{g}}}_1\oslash {}^\gamma {{\dot{g}}}_3) \end{aligned}$$

#### Proof

We can deduce from the definitions of the operators (4) and (7) given that

$$\begin{aligned}&({}^\gamma {{\dot{g}}}_1\oplus {}^\gamma {{\dot{g}}}_2)\ominus ({}^\gamma {{\dot{g}}}_2\oplus {}^\gamma {{\dot{g}}}_3)\\&\quad = (\{\langle 1-(1-{{\dot{\textit{g} }}^1_1})(1-{{\dot{\textit{g} }}^1_2}) ,{{\gamma }_*^{1}}\rangle ,\ldots ,\langle 1-(1-{{\dot{\textit{g} }}^{p^*}_1})(1-{{\dot{\textit{g} }}^{p^*}_2}) ,{{\gamma }_*^{p^*}}\rangle \})\\&\qquad \ominus (\{\langle 1-(1-{{\dot{\textit{g} }}^1_2})(1-{{\dot{\textit{g} }}^1_3}) ,{{\gamma }_*^{1}}\rangle ,\ldots ,\langle 1-(1-{{\dot{\textit{g} }}^{p^*}_2})(1-{{\dot{\textit{g} }}^{p^*}_3}) ,{{\gamma }_*^{p^*}}\rangle \})\\&\quad = \left\{ \left\langle \max \left\{ 0,\frac{ [1-(1-{{\dot{\textit{g} }}^1_1})(1-{{\dot{\textit{g} }}^1_2})] - [1-(1-{{\dot{\textit{g} }}^1_2})(1-{{\dot{\textit{g} }}^1_3})] }{1- [1-(1-{{\dot{\textit{g} }}^1_2})(1-{{\dot{\textit{g} }}^1_3})] } ,\gamma ^{1}_*\right\rangle ,\right. \right. \\&\qquad \ldots , \left\langle \max \left\{ 0,\frac{ [1-(1-{{\dot{\textit{g} }}^{p^*}_1})(1-{{\dot{\textit{g} }}^{p^*}_2})] - [1-(1-{{\dot{\textit{g} }}^{p^*}_2})(1-{{\dot{\textit{g} }}^{p^*}_3})] }{1- [1-(1-{{\dot{\textit{g} }}^{p^*}_2})(1-{{\dot{\textit{g} }}^{p^*}_3})] } ,\gamma ^{p^*}_*\right\rangle \right\} \\&\quad = \left\{ \left\langle \max \left\{ 0,\frac{ -(1-{{\dot{\textit{g}}}^1_1})(1-{{\dot{\textit{g} }}^1_2})+(1-{{\dot{\textit{g}}}^1_2}) (1-{{\dot{\textit{g} }}^1_3})}{(1-{{\dot{\textit{g}}}^1_2})(1-{{\dot{\textit{g} }}^1_3}) } ,\gamma ^{1}_*\right\rangle ,\right. \right. \\&\qquad \ldots , \left\langle \max \left\{ 0,\frac{ (1-{{\dot{\textit{g} }}^{p^*}_1})(1-{{\dot{\textit{g}}}^{p^*}_2}) +(1-{{\dot{\textit{g} }}^{p^*}_2})(1-{{\dot{\textit{g} }}^{p^*}_3}) }{(1-{{\dot{\textit{g} }}^{p^*}_2})(1-{{\dot{\textit{g} }}^{p^*}_3}) } ,\gamma ^{p^*}_*\right\rangle \right\} \\&\quad = \left\{ \left\langle \max \left\{ 0,\frac{ {{\dot{\textit{g} }}^1_1}-{{\dot{\textit{g} }}^1_3} }{1-{{\dot{\textit{g} }}^1_3} } ,\gamma ^{1}_*\right\rangle ,\ldots , \left\langle \max \left\{ 0,\frac{ {{\dot{\textit{g} }}^{p^*}_1}-{{\dot{\textit{g} }}^{p^*}_3} }{1-{{\dot{\textit{g} }}^{p^*}_3} } ,\gamma ^{p^*}_*\right\rangle \right\} \right. \right. \\&\quad ={}^\gamma {{\dot{g}}}_1\ominus {}^\gamma {{\dot{g}}}_3 \end{aligned}$$

We can deduce from the definitions of the operators (5) and (6) that

$$\begin{aligned}&({}^\gamma {{\dot{g}}}_1\otimes {}^\gamma {{\dot{g}}}_2)\oslash ({}^\gamma {{\dot{g}}}_2\otimes {}^\gamma {{\dot{g}}}_3)\\&\quad = (\{\langle {{\dot{\textit{g} }}^1_1}{{\dot{\textit{g} }}^1_2} ,{{\gamma }_*^{1}}\rangle ,\ldots ,\langle {{\dot{\textit{g} }}^{p^*}_1}{{\dot{\textit{g} }}^{p^*}_2} ,{{\gamma }_*^{p^*}}\rangle \})\\&\qquad \oslash (\{\langle {{\dot{\textit{g} }}^1_2}{{\dot{\textit{g} }}^1_3} ,{{\gamma }_*^{1}}\rangle ,\ldots ,\langle {{\dot{\textit{g} }}^{p^*}_2}{{\dot{\textit{g} }}^{p^*}_3} ,{{\gamma }_*^{p^*}}\rangle \})\\&\quad = \left\{ \left\langle \min \left\{ 1, \frac{{{\dot{\textit{g} }}^1_1}{{\dot{\textit{g}}}^1_2}}{{{\dot{\textit{g} }}^1_2}{{\dot{\textit{g} }}^1_3}}\right\} ,\gamma ^{1}_*\right\rangle ,\ldots , \left\langle \min \left\{ 1, \frac{{{\dot{\textit{g} }}^{p^*}_1}{{\dot{\textit{g} }}^{p^*}_2}}{{{\dot{\textit{g} }}^{p^*}_2}{{\dot{\textit{g} }}^{p^*}_3}}\right\} ,\gamma ^{{p^*}}_*\right\rangle \right\} \\&\quad = \left\{ \left\langle \min \left\{ 1, \frac{{{\dot{\textit{g}}}^1_1}}{{{\dot{\textit{g} }}^1_3}}\right\} ,\gamma ^{1}_*\right\rangle ,\ldots , \left\langle \min \left\{ 1, \frac{{{\dot{\textit{g} }}^{p^*}_1}}{{{\dot{\textit{g} }}^{p^*}_3}}\right\} ,\gamma ^{{p^*}}_*\right\rangle \right\} \\&\quad = {}^\gamma {{\dot{g}}}_1\oslash {}^\gamma {{\dot{g}}}_3 \end{aligned}$$

## Proposed methodology

**Figure 4:**
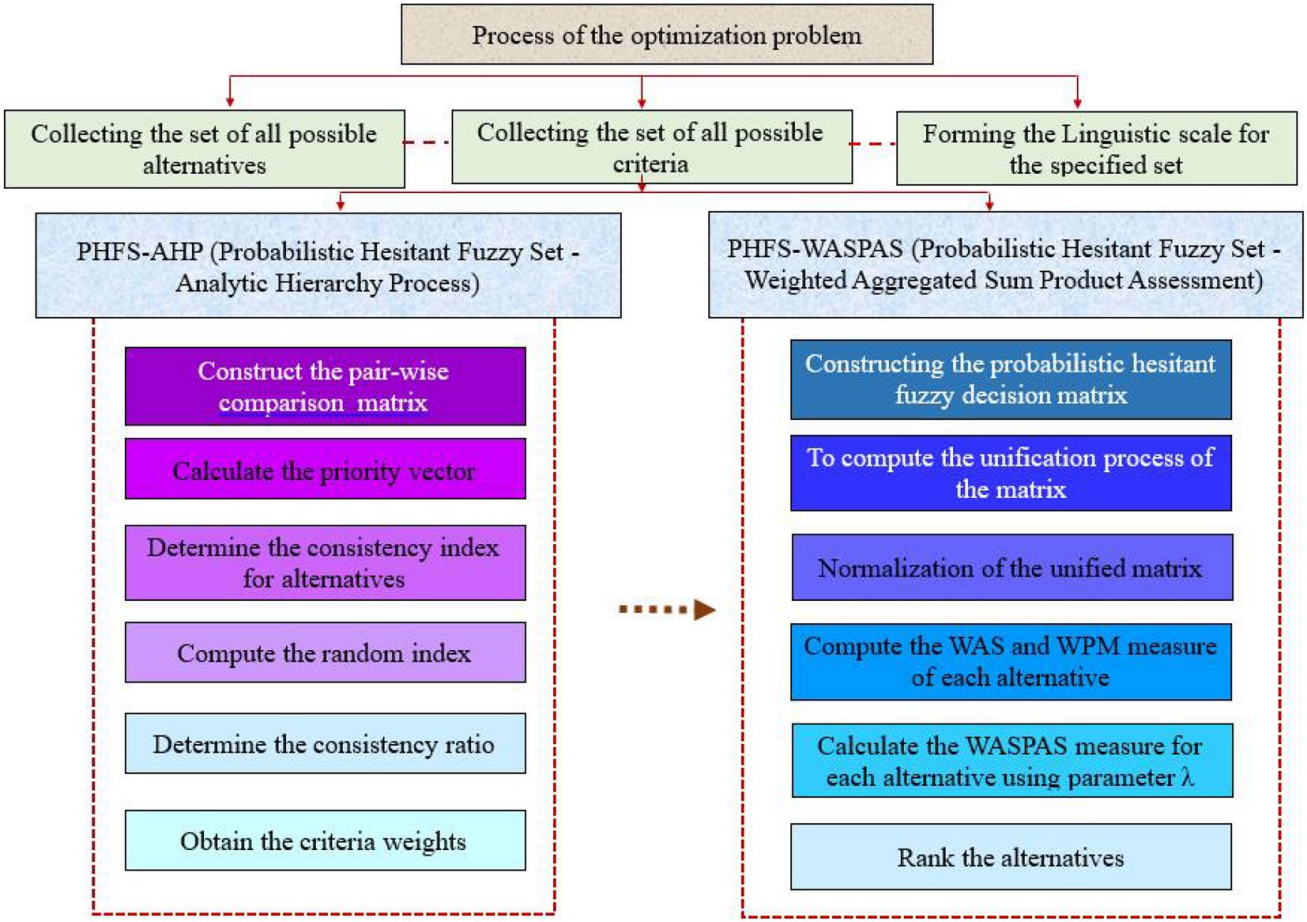
A flowchart that depicts the steps involved in solving the optimization problem.

The probabilistic hesitant fuzzy based AHP method is incorporated for determining the crieria weights and the WASPAS method is used for prioritizing the alternatives. The framework of the proposed method is shown in Fig. 4.

### Algorithm of PHFS based AHP method

Complex judgments are broken down into a series of pairwise comparisons using AHP. The resulting pairwise comparison matrix is obtained in the form,

$$\begin{aligned} {\mathbb {A}}= \left[ \begin{array}{cccc} 1 &{} {\mathbb {A}}_{12} &{} \cdots &{} {\mathbb {A}}_{1 n} \\ \vdots &{} \vdots &{} \vdots &{} \vdots \\ {\mathbb {A}}_{n1} &{} {\mathbb {A}}_{n2} &{} \cdots &{} 1 \end{array}\right] . \end{aligned}$$

*Step 1* Normalized matrix Divide each entry in the pairwise comparison matrix by its corresponding column sum.

*Step 2* Priority vector An average of the row items in the normalized matrix yields the priority vector. Converting to decimals.

*Step 3* The weighted sum vector’s components are separated into groups based on the importance of each criterion. The values’ average is then calculated and denoted by $\lambda _{max}$.

*Step 4* Determine the consistency index, CI, of the $\textrm{n}$ alternatives by:

$$\begin{aligned} C I=\left( \lambda _{\max }-n\right) /(n-1) \end{aligned}$$

*Step 5* Compute the random index, RI, as follows: Table 2:

**Table 2:** The values of the Random Index.

Number of alternatives (n)	Random index (RI)	Number of alternatives (n)	Random index (RI)
3	0.58	6	1.24
4	0.9	7	1.32
5	1.12	8	1.41

*Step 6* Determine the consistency ratio $\textrm{CR}=\textrm{CI} / \textrm{RI}$. If $C I / R I<0.1$ the degree of consistency is acceptable, otherwise AHP is not meaningful.

### Algorithm of PHFS based WASPAS method

Now evaluate the following decision matrix, in which the elements are PHFEs:

$$\begin{aligned} D_{ij}= \left[ \begin{array}{cccc} &{}{}P_1&{}{} P_2&{}{} \cdots P_n\\ Q_1&{}{} {}^\gamma {g}_{11}&{}{} {}^\gamma {g}_{12}&{}{}{}^\gamma {g}_{1n}\\ Q_2&{}{} {}^\gamma {g}_{21}&{}{} {}^\gamma {g}_{22}&{}{}{}^\gamma {g}_{2n}\\ \vdots &{}{}\vdots &{}{}\vdots &{}{}\vdots \\ Q_m&{}{} {}^\gamma {g}_{m1}&{}{} {}^\gamma {g}_{m2}&{}{}{}^\gamma {g}_{mn}\\ \end{array} \right] \end{aligned}$$

where ${}^\gamma {g}_{ij}$ indicates the score of alternative $Q_i$ comparable to the criteria $P_j$ with the weight of $w_j$. We’ll unify all of the entries in the decision matrix D to get ${}^\gamma {{\dot{g}}}_{ij}=\bigcup _{\langle {{\dot{\textit{g} }}_{ij}} ,{{\dot{\gamma }}}_{ij}\rangle \in {}^\gamma {{\dot{g}}}_{ij}}\{\langle {{\dot{\textit{g} }}_{ij}},{{\dot{\gamma }}}_{ij}\rangle \}$ as described in the before section.

*Step 1* Decision matrix is normalized. In cases where the criteria are based on cost and beneficial, $D_{ij}$ is utilized,

8 $$\begin{aligned}&{{\overline{D}}}_{ij}= \left\{ \begin{array}{ll} D_{ij}\oslash \max _i\{D_{ij}\} &{}{} \quad {\textit{for beneficial criteria}} \\ \min _i\{D_{ij}\}\oslash D_{ij} &{}{} \quad {\textit{for cost criteria}} \end{array} \right. \end{aligned}$$

*Step 2* The WSM method is used to calculate the performance of alternative Eq. (9) below, where $w_j$ represents the criterion weights.

9 $$\begin{aligned} S_{i}^{(1)}=\sum _{j=1}^{n}{{\overline{D}}}_{ij} w_{j} \end{aligned}$$

*Step 3* The WPM method calculates the performance characteristics of alternatives; Eq. (10) is used to express this.

10 $$\begin{aligned} S_{i}^{(2)}=\prod _{j=1}^{n}\left( {{\overline{D}}}_{ij}\right) ^{w_{j}} \end{aligned}$$

*Step 4* Alternatives’ final performance $S_i$ represents the postures of alternatives in a broad ranking and is calculated by adding the variety of data derived by Eqs. (9) and (10). $\alpha$ is a parameter with values ranging from 0 to 1.

11 $$\begin{aligned} S_{i}=\alpha S_{i}^{(1)} + (1-\alpha ) S_{i}^{(2)} \end{aligned}$$

*Step 5* Alternatives are ranked.To determine the final rankings of alternatives, $S_i$ values are ranked from highest to lowest.

## Case study

**Figure 5:**
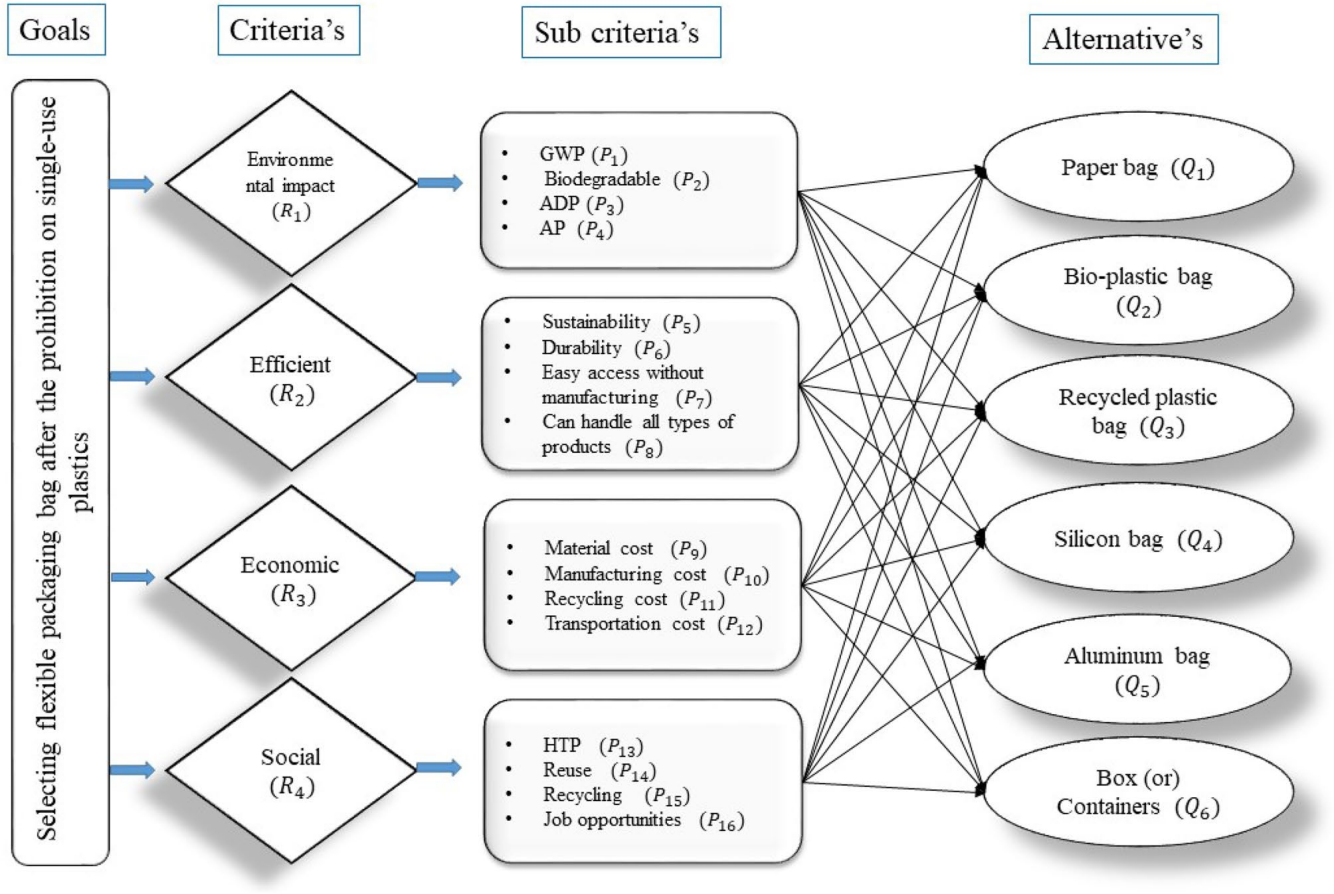
The hierarchical organization of the chosen criteria.

This research study sought to investigate and propose alternatives to plastic bags and boxes that do not jeopardise citizens’ life styles. The Fuzzy WASPAS-MCDM technique for comparison and prioritisation of alternatives. The six alternate solution bags are evaluated in terms of multiple dimensions of sustainability, as well as an assessment of biodegradable plastic bags. According to the results of the analysis, producing bio-plastic bottles instead of biodegradable polymer bags would result in a reduction of negative impacts on the environment. UN Environment Programme (UNEP) defines single-use plastic products as “an umbrella term for different types of products that are typically used once before being thrown away or recycled” (UNEP 2018), which includes food packaging, bottles, straws, containers, cups, cutlery, and shopping bags. According to estimates, 8 million metric tonnes of plastic are discarded into the oceans annually, and between 100 and 150 million metric tonnes of plastic are produced for single-use products (Plastics Oceans 2019). Alternatives to single-use plastic products must be taken into account. Addressing Single-Use Plastic Products Pollution, Resolution 9 of the Fourth United Nations Environment Assembly (UNEA4) in March 2019 (UNEP/EA.4/R.9) “encourages member states to take appropriate actions to promote the identification and development of environmentally friendly alternatives to single-use plastic products, taking into account the full life cycle implications of those alternatives.” Here we considered these six types of alternative bags; $Q_1$: Paper bag, $Q_2$: Bio-plastic bag, $Q_3$: Recycled plastic bag, $Q_4$: Silicon bag, $Q_5$: Aluminum (or) metal based bag, $Q_6$: Box(or)containers these six alternative choices, based on the following four main criteria.This research conversation aimed to recommend plastic pollution prevention measures by analysing alternative approaches for packaging plastic bags and boxes show in Fig. 5.

- *Paper bag*($Q_1$) Materials are commonly used to make wrapping paper for fast food packaging. Raw material is used in the production of paper-based wrapping paper. Greaseproof paper, wax coated paper, and foil papers are examples of specialised papers, such as kraft paper. Paper bags are reusable, recyclable, and biodegradable. aids in the conservation of marine wildlife Young children and animals are less likely to suffocate when using paper bags.
- *Bio-plastic bag* $(Q_2)$ Bio-based plastics are created entirely (or in part) from biological resources instead of fossil-based basic materials. Compostable (or biodegradable) is not a requirement. Plant-based plastics are known as bio-plastics. In every case, these bags are less harmful to the environment. Green and recyclable bio-bags are the two types of bio-bags we offer.
- *Recycled plastic bag* $(Q_3)$ Plastic can take 400–1000 years to decompose naturally, and some forms of plastic are non degradable. Although plastic has many use, its environmental and health risks are not insignificant. When plastic bags are burned, they emit hazardous vapours and take thousands of years to decompose. Then, when it’s time to release greenhouse gases, when they are revealed to ultraviolet radiation.
- *Silicon bag* $(Q_4)$ At the store, place your food in thick airtight silicon food storage bags, refrigerate (or) freeze , marinade , microwave , or boil . These reusable freezer bags are durable, flexible, and washable. The bags are constructed of food-grade silicone manufactured from silico sand of the finest quality. The bags are leak-proof and airtight thanks to the top sealing bar, which is made of strong plastic.
- *Aluminum (or) metal based bag* $(Q_5)$ Foils bags—High barrier packaging metallic bags, often known as foil bags, are more than just gleaming parcels. These metallic bags feature high barrier capabilities and are made of a light weight, durable material, making them an appealing flexible packaging solution for a number of uses.
- *Box (or) containers* $(Q_6)$ Plastic containers are still one of the most popular ways to classify and move goods that you own (or create). High-strength polypropylene is used to make plastic storage containers, with some even being recycled. Plastic may (or may not) be the best option.

### Criteria weight evaluation using PHFS-AHP

The pairwise comparison methodology described is used to accomplish all of these. Pair-wise comparison matrix is created with the help of the scale of relative importance shown in Table 3.

**Table 3:** The values of Random Index.

Saaty’s scale	Description
1	Equal importance
3	Moderate importance
5	Strong importance
7	Very strong importance
9	Extreme importance
2, 4, 6, 8	Intermediate values
1/3, 1/5, 1/7, 1/9	Values for inverse comparison

**Figure 6:**
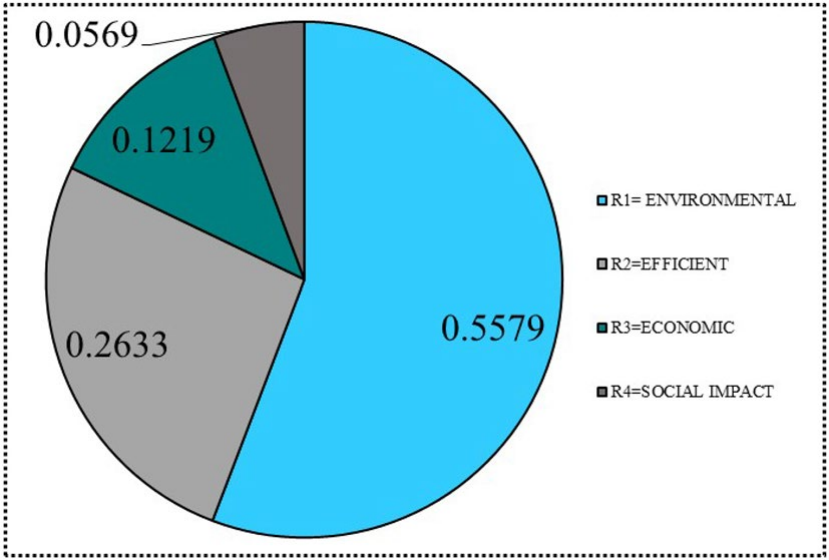
Weights of the main criteria.

For instance, the five criteria are evaluated by comparing them in pairs with the goal in order to determine the relative importance of each criteria to the goal. Table 4 depicts a pairwise comparison of all criteria in order to determine the weighting of each criterion in the decision-making process as shown in Fig. 6.

**Table 4:** A pairwise comparison of the main criteria.

	Environment impact	Efficient	Economic	Social
Environment impact	1	3	5	7
Efficient	1/3	1	3	5
Economic	1/5	1/3	1	3
Social	1/5	1/5	1/3	1

Finally, by averaging all rows, the priorities for the criterion as eigenvector (weights of criteria) ${\mathbb {X}}$ can be determined, as shown below:

$$\begin{aligned} \begin{array}{c|c|} &{} 0.5578 \\ &{} 0.2633 \\ {\mathbb {X}} = &{} 0.1218 \\ &{} 0.0568 \end{array} \end{aligned}$$

As shown by eigenvector ${\mathbb {X}}$, the interest expense was determined to be the least important criterion with a value of 0.0568, while the number of crews was determined to be the most relevant criterion with a value of 0.5578.

**Figure 7:**
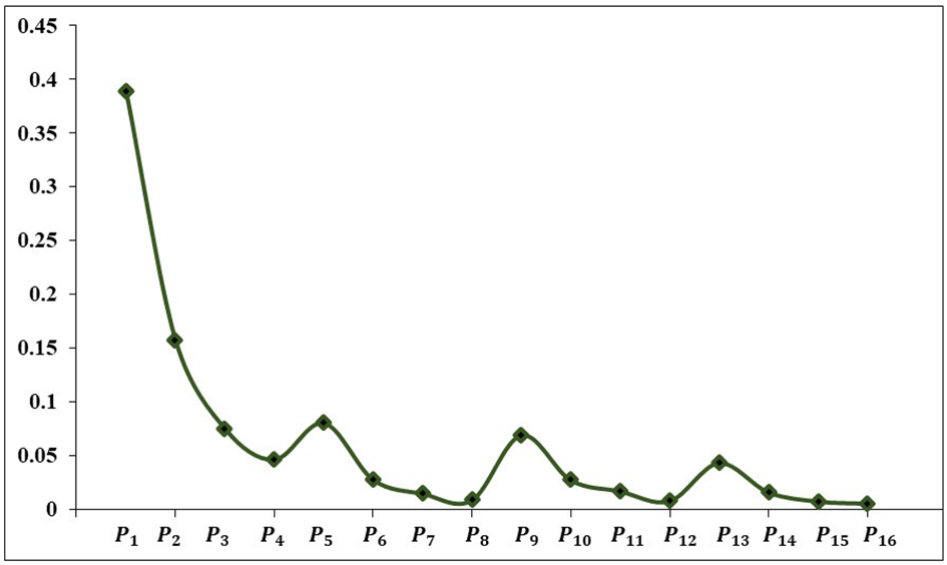
Weights of the sub-criteria.

Then, by calculating CI and CR values, the consistency of decision-makers’ judgments is examined. The calculated CI and CR values are 0.0394 and 0.0438, respectively. Because their CR values are less than 0.1, decision-makers’ assessments are reliable. As a result, the pairwise comparison of each sub-criteria weight is a local weight. Finally, a complete set of eventual weighting scores for each alternative is then secured by linear multiplication of the global weight as shown in the Fig. 7.

### The proposed method consist of fuzzy PHFEs-WASPAS

Now, unified of ${}^\gamma {{\dot{g}}}_{ij}$, how to compare qualitative and quantitative data? Prioritizing the factors (criteria) affecting the goal of decision making and selecting the best alternative by using the linguistic conversion scale Table 5.

**Table 5:** Linguistic conversion scale with corresponding PHFEs.

Linguistic term	Numerical values with PHFEs
Very highest	{$\langle 0.8, 0.3 \rangle$} − {$\langle 1.0, 0.7 \rangle$}
Highest	{$\langle 0.7, 0.5 \rangle$} − {$\langle 0.8, 0.5 \rangle$}
Moderate	$\{\langle 0.5, 0.6 \rangle$} − {$\langle 0.7, 0.4 \rangle$}
Lowest	{$\langle 0.3, 0.3 \rangle$} − {$\langle 0.5, 0.8 \rangle$}
Very lowest	{$\langle 0.1, 0.2 \rangle$} − {$\langle 0.3, 0.8 \rangle$}

The experts are allowed to express their aspirations for the preceding plastic bags using PHFEs, which are summarised in the PHF decision matrix shown below (Table 5).

We combine all of the records in the decision matrix D once the algorithm is applied (Table 6). We get ${}^\gamma {{\dot{g}}}_{ij} , (i=1,\ldots ,m, j=1,\ldots ,n)$ as previously mentioned. Hence, we will work on the UPHFEs in the future. ${}^\gamma {{\dot{g}}}_{ij}=\bigcup _{\langle {{\dot{\textit{g}}}_{ij}} ,{{\dot{\gamma }}}_{ij}\rangle \in {}^\gamma {{\dot{g}}}_{ij}}\{\langle {{\dot{\textit{g} }}_{ij}},{{\dot{\gamma }}}_{ij}\rangle \}$ (using Figs. 1,2 and 3 model of unification procedure, then we will get Table 7).

*Step 1*

The normalized score decision matrix as the following Eq. (8).

Then the following division operation on two PHFEs, can be introduced

$$\begin{aligned} {}^\gamma {{\dot{g}}}_1\oslash {}^\gamma {{\dot{g}}}_2=&{} \left\{ \left\langle \min \left\{ 1,\frac{{{\dot{\textit{g} }}^{1}_1}}{{{\dot{\textit{g} }}^{1}_2}}\right\} ,\gamma ^{1}_*\right\rangle ,\ldots \left\langle \min \left\{ 1,\frac{{{\dot{\textit{g} }}^{p^*}_1}}{{{\dot{\textit{g}}}^{l^*}_2}}\right\} ,\gamma ^{p^*}_*\right\rangle \right\} \\&\gamma ^1_{*}=\min \{\gamma ^{1}_1,\gamma ^{1}_2,\ldots ,\gamma ^{1}_m\} \end{aligned}$$

The result of values shown in Table 8.

*Step 2* The WSM method is used to calculate the performance of alternative Eq. (9). The result of values shown in Table 9.

$$S_{i}^{(1)}$$={0.773531767, 0.813966797, 0.273892414, 0.65780551, 0.485707069, 0.377106607}

*Step 3* The WPM method calculates the performance characteristics of alternatives; Eq. (10) is used to the result of values shown in Table 10.

$$S_{i}^{(2)}$$={0.730946078, 0.747884875, 0.262539502, 0.649503081, 0.453691938, 0.339879367}

*Step 4* Alternatives’ final performance $S_i$ represents the postures of alternatives in a broad ranking and is calculated by adding the variety of data derived by Eqs. (9) and (10). $\alpha$ is a parameter with values ranging from 0 to 1.

$$S_{i}$$={0.752238922, 0.780925836, 0.268215958, 0.653654296, 0.469699503, 0.358492987}

*Step 5* Alternatives are ranked. The $Q_2$ alternative is the right choice in this ranking, while the $Q_3$ alternative is by far the worst. Rank all of the alternatives $Q_i$ that correspond to the sequence of $S_i$. Also, the results are shown in Fig. 10.

$$\begin{aligned} Q_{2}> Q_{1}> Q_{4}> Q_{5}> Q_{6} > Q_{3} \end{aligned}$$

## Result and discussion

**Figure 8:**
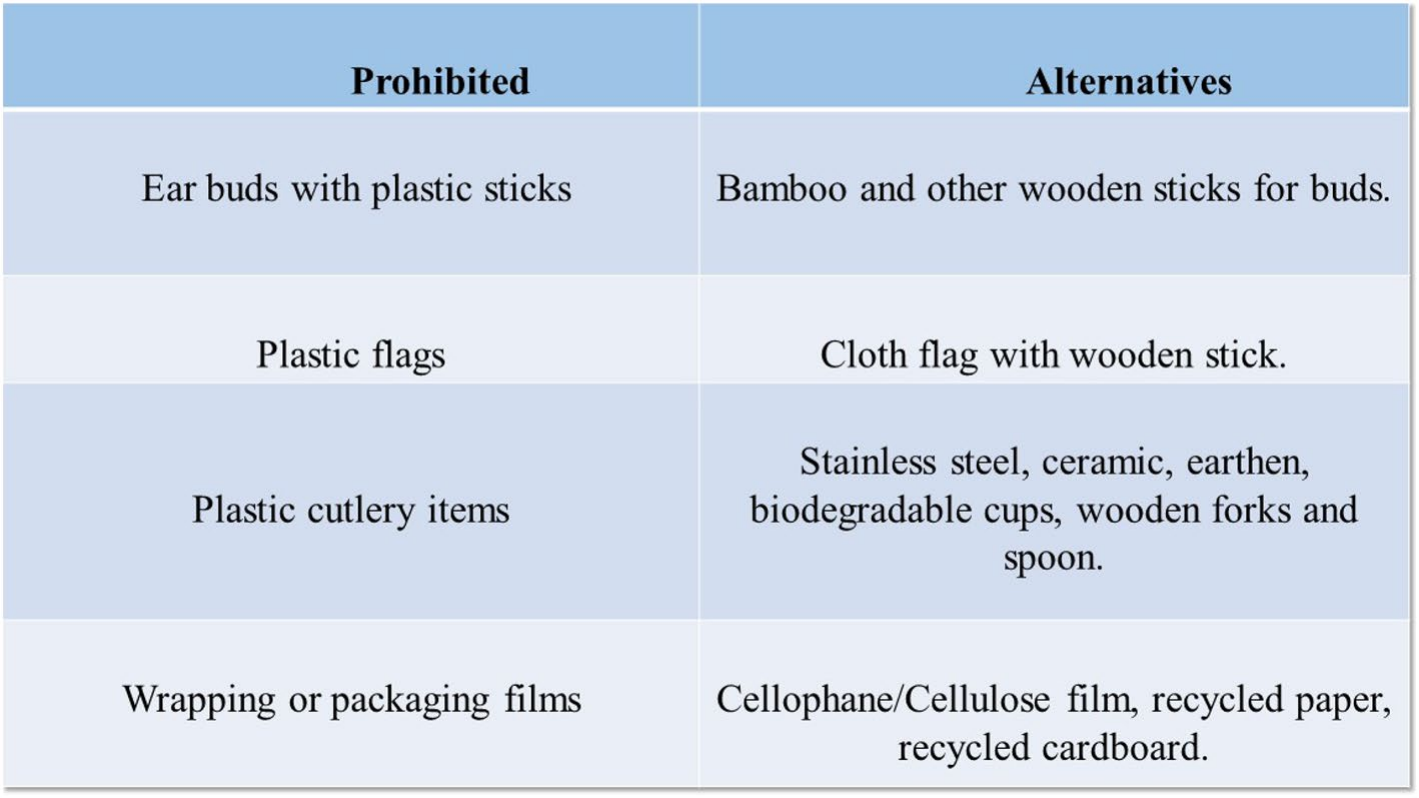
Efforts for a plastic-free society.

The goal of this paper discussion was to propose plastic prevention measures for packaging products and plastic containers using performance alternatives. We used PHFS in the AHP weight-finding strategy, which is an essential feature of the MCDM method. As a result, we have included the PHFS-WASPAS in our selection of the most effective hesitant fuzzy environment for bio-plastic bags. The ranking results for the suggested PHFS-MCDM techniques surpassed the existing AHP methods in the research study by providing the best solution. The PHFS-WASPAS method had to consider four main criteria, as well as sixteen sub-criteria, including alternative bags are paper bags $(Q_1)$, bio-plastic bags $(Q_2)$, recycled plastic bags $(Q_3)$, silicon bags $(Q_4)$, aluminum bags $(Q_5)$, and boxes or containers $(Q_6)$. After applying the MCDM methodology, this bio-plastic bag proved to be the most effective flexible packaging bag after the prohibition on single-use plastics. The fact that bio-plastic bags have almost all of the desirable properties throughout the manufacturing process, including very low human toxicity, medium (greenhouse gas) emissions, and biodegradability could be compared to more results. People are becoming more aware of global warming, according to the analysis of fuzzy WASPAS results. Additionally, no impact category particularly connected to plastic pollution was included in any of the earlier research on shopping bags. To account for plastic pollution in the environment (and material pollution in general), there has been a methodological gap to date. Plastic bags are the fifth most often collected item worldwide, according to studies that provide an overview of the level of leakage and plastic pollution through beach clean-ups. A significant amount of plastic pollution in the marine environment originates locally in coastal cities and towns, according to other research that has looked into its sources and paths. Beach surveys have recently been used to calculate the rates at which marine debris accumulates and the propensity of items to leach into the ecosystem. The traditional set of LCIA mid-point effect categories has been expanded in this study to include a new indication called persistence. The indicator takes into account waste disposal, leakage, and bio degradation to account for the effects of material contamination in the receiving environment. As anticipated, the single-use plastic bags had the highest persistence, followed by the re-usable petro-based bags and biodegradable (either petro- or bio-based) bags with the lowest persistence impacts and efforts for a plastic-free society as shown in the Fig. 8.

**Figure 9:**
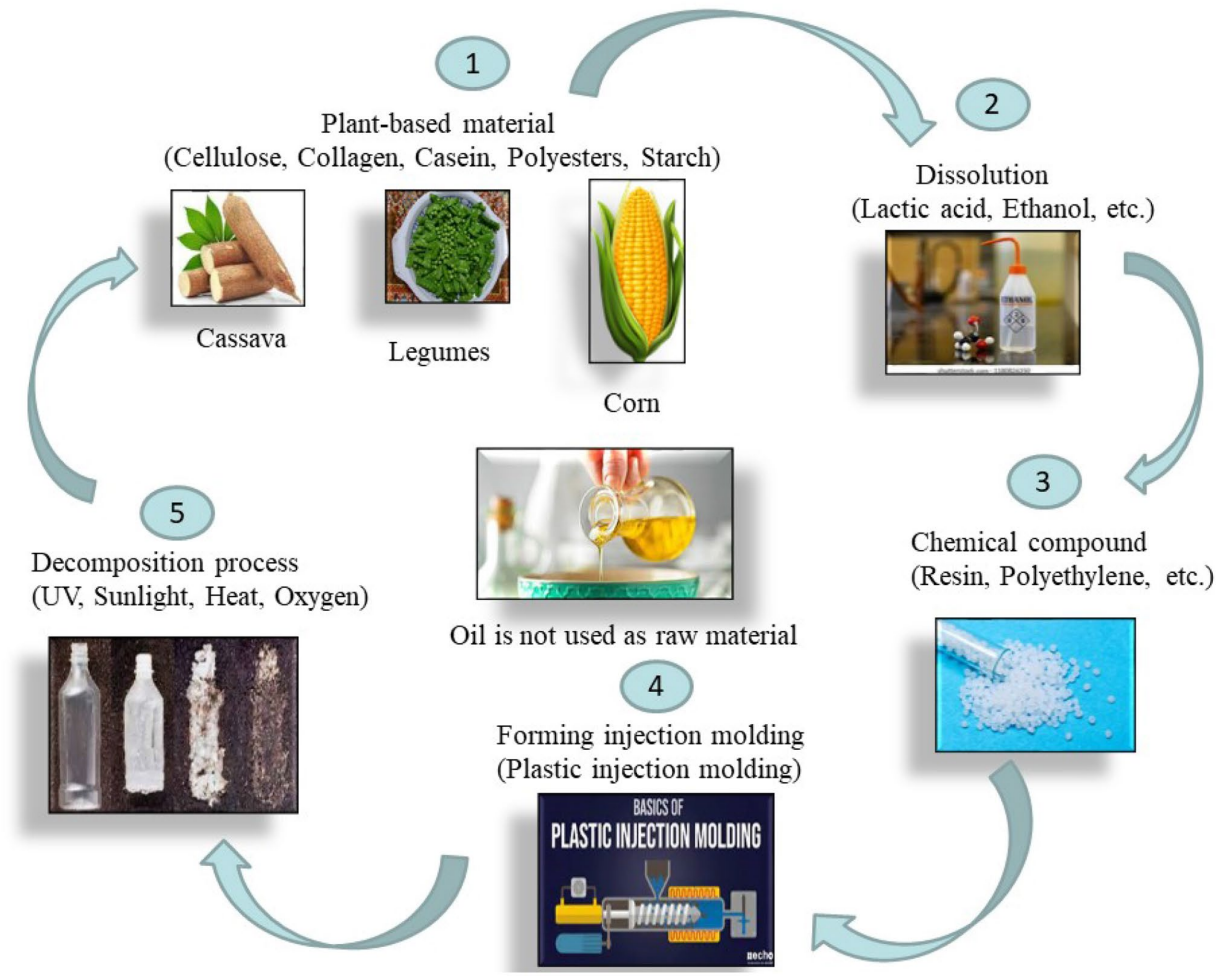
Bio-plastic.

Although the inclusion of an ingredient to speed up the bio degradation of petro-based HDPE reduced the persistence, it was still at least a factor of ten higher than that of the biodegradable bags (paper, bio plastic, and silicon), and the manufacturer’s claims about the bags’ authenticity have been questioned (ECM biofilms 2021). Given their high rates of bio degradation, the results of the persistent indicate that bio plastic bags and paper bags (PBAT+Starch and PBS+PBAT) have the lowest material pollution impacts, followed by reusable bag solutions that prevent the production and use of materials altogether as shown in the Fig. 9.

**Figure 10:**
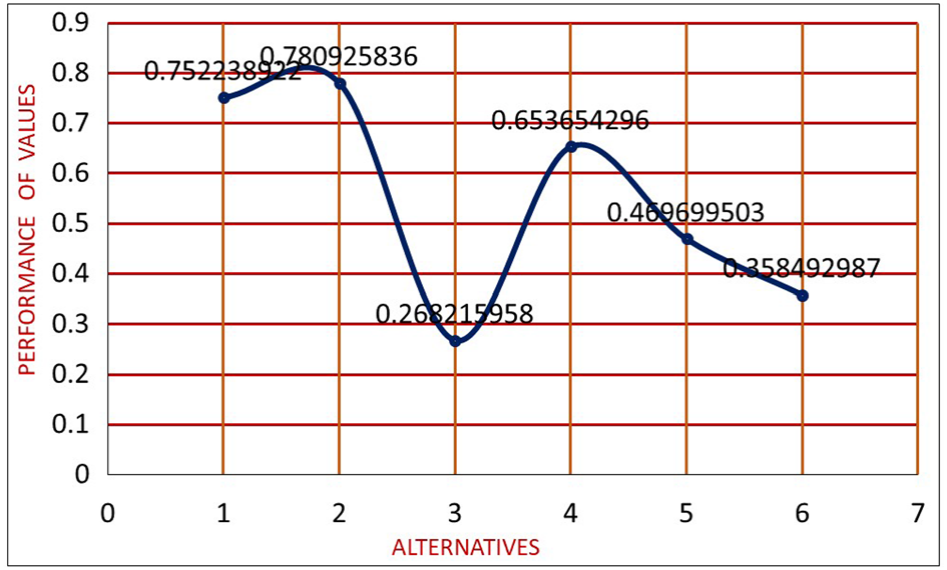
The proposed WASPAS method.

**Table 6:** The probabilistic hesitant fuzzy decision matrix.

	$P_1$	$P_2$	$P_3$	$P_4$	$P_5$	$P_6$	$P_7$	$P_8$
$Q_1$	{$\langle 0.2,0.5\rangle ,\langle 0.3,0.5\rangle$}	{$\langle 0.1,0.5\rangle ,\langle 0.2,0.5\rangle$}	{$\langle 0.1,0.3\rangle ,\langle 0.2,0.7\rangle$}	{$\langle 0.2,0.4\rangle ,\langle 0.3,0.6\rangle$}	{$\langle 0.7,0.5\rangle ,\langle 0.8,0.5\rangle$}	{$\langle 0.1,0.5\rangle ,\langle 0.2,0.5\rangle$}	{$\langle 0.4,0.4\rangle ,\langle 0.5,0.6\rangle$}	{$\langle 0.4,0.3\rangle ,\langle 0.5,0.7\rangle$}
$Q_2$	{$\langle 0.1,0.3\rangle ,\langle 0.2,0.7\rangle$}	{$\langle 0.2,0.5\rangle ,\langle 0.3,0.5\rangle$}	{$\langle 0.2,0.2\rangle ,\langle 0.3,0.8\rangle$}	{$\langle 0.1,0.5\rangle ,\langle 0.2,0.5\rangle$}	{$\langle 0.8,0.3\rangle ,\langle 0.9,0.7\rangle$}	{$\langle 0.3,0.3\rangle ,\langle 0.4,0.7\rangle$}	{$\langle 0.5,0.3\rangle ,\langle 0.6,0.7\rangle$}	{$\langle 0.6,0.5\rangle ,\langle 0.7,0.5\rangle$}
$Q_3$	{$\langle 0.6,0.5\rangle ,\langle 0.7,0.5\rangle$}	{$\langle 0.7,0.7\rangle ,\langle 0.8,0.3\rangle$}	{$\langle 0.5,0.3\rangle ,\langle 0.6,0.7\rangle$}	{$\langle 0.6,0.4\rangle ,\langle 0.7,0.6\rangle$}	{$\langle 0.1,0.4\rangle ,\langle 0.2,0.6\rangle$}	{$\langle 0.3,0.8\rangle ,\langle 0.4,0.2\rangle$}	{$\langle 0.1,0.5\rangle ,\langle 0.2,0.5\rangle$}	{$\langle 0.1,0.4\rangle ,\langle 0.2,0.6\rangle$}
$Q_4$	{$\langle 0.2,0.5\rangle ,\langle 0.3,0.5\rangle$}	{$\langle 0.2,0.2\rangle ,\langle 0.3,0.8\rangle$}	{$\langle 0.2,0.6\rangle ,\langle 0.3,0.4\rangle$}	{$\langle 0.2,0.5\rangle ,\langle 0.3,0.5\rangle$}	{$\langle 0.6,0.2\rangle ,\langle 0.7,0.8\rangle$}	{$\langle 0.7,0.3\rangle ,\langle 0.8,0.7\rangle$}	{$\langle 0.3,0.8\rangle ,\langle 0.4,0.2\rangle$}	{$\langle 0.4,0.7\rangle ,\langle 0.5,0.3\rangle$}
$Q_5$	{$\langle 0.4,0.3\rangle ,\langle 0.5,0.7\rangle$}	{$\langle 0.3,0.4\rangle ,\langle 0.4,0.6\rangle$}	{$\langle 0.3,0.7\rangle ,\langle 0.4,0.3\rangle$}	{$\langle 0.3,0.3\rangle ,\langle 0.4,0.7\rangle$}	{$\langle 0.4,0.5\rangle ,\langle 0.5,0.5\rangle$}	{$\langle 0.6,0.4\rangle ,\langle 0.7,0.6\rangle$}	{$\langle 0.2,0.5\rangle ,\langle 0.3,0.5\rangle$}	{$\langle 0.5,0.4\rangle ,\langle 0.6,0.6\rangle$}
$Q_6$	{$\langle 0.5,0.2\rangle ,\langle 0.6,0.8\rangle$}	{$\langle 0.5,0.3\rangle ,\langle 0.6,0.7\rangle$}	{$\langle 0.6,0.5\rangle ,\langle 0.7,0.5\rangle$}	{$\langle 0.5,0.2\rangle ,\langle 0.6,0.8\rangle$}	{$\langle 0.3,0.6\rangle ,\langle 0.4,0.4\rangle$}	{$\langle 0.5,0.5\rangle ,\langle 0.6,0.5\rangle$}	{$\langle 0.2,0.6\rangle ,\langle 0.3,0.4\rangle$}	{$\langle 0.4,0.8\rangle ,\langle 0.5,0.2\rangle$}

	$P_9$	$P_{10}$	$P_{11}$	$P_{12}$	$P_{13}$	$P_{14}$	$P_{15}$	$P_{16}$
$Q_1$	{$\langle 0.3,0.8\rangle ,\langle 0.4,0.2\rangle$}	{$\langle 0.7,0.2\rangle ,\langle 0.8,0.8\rangle$}	{$\langle 0.2,0.3\rangle ,\langle 0.3,0.7\rangle$}	{$\langle 0.3,0.4\rangle ,\langle 0.4,0.6\rangle$}	{$\langle 0.7,0.5\rangle ,\langle 0.8,0.5\rangle$}	{$\langle 0.6,0.5\rangle ,\langle 0.7,0.5\rangle$}	{$\langle 0.7,0.7\rangle ,\langle 0.8,0.3\rangle$}	{$\langle 0.4,0.2\rangle ,\langle 0.5,0.8\rangle$}
$Q_2$	{$\langle 0.5,0.2\rangle ,\langle 0.6,0.8\rangle$}	{$\langle 0.8,0.8\rangle ,\langle 0.9,0.2\rangle$}	{$\langle 0.1,0.2\rangle ,\langle 0.2,0.8\rangle$}	{$\langle 0.8,0.3\rangle ,\langle 0.9,0.7\rangle$}	{$\langle 0.6,0.5\rangle ,\langle 0.7,0.5\rangle$}	{$\langle 0.7,0.2\rangle ,\langle 0.8,0.2\rangle$}	{$\langle 0.8,0.3\rangle ,\langle 0.9,0.7\rangle$}	{$\langle 0.6,0.4\rangle ,\langle 0.7,0.6\rangle$}
$Q_3$	{$\langle 0.1,0.5\rangle ,\langle 0.2,0.5\rangle$}	{$\langle 0.2,0.2\rangle ,\langle 0.3,0.8\rangle$}	{$\langle 0.4,0.4\rangle ,\langle 0.5,0.6\rangle$}	{$\langle 0.2,0.6\rangle ,\langle 0.3,0.4\rangle$}	{$\langle 0.1,0.5\rangle ,\langle 0.2,0.5\rangle$}	{$\langle 0.1,0.6\rangle ,\langle 0.2,0.4\rangle$}	{$\langle 0.4,0.5\rangle ,\langle 0.6,0.5\rangle$}	{$\langle 0.2,0.3\rangle ,\langle 0.3,0.7\rangle$}
$Q_4$	{$\langle 0.2,0.6\rangle ,\langle 0.3,0.4\rangle$}	{$\langle 0.2,0.3\rangle ,\langle 0.3,0.7\rangle$}	{$\langle 0.3,0.7\rangle ,\langle 0.4,0.3\rangle$}	{$\langle 0.3,0.4\rangle ,\langle 0.4,0.6\rangle$}	{$\langle 0.5,0.7\rangle ,\langle 0.6,0.3\rangle$}	{$\langle 0.1,0.3\rangle ,\langle 0.2,0.7\rangle$}	{$\langle 0.7,0.2\rangle ,\langle 0.8,0.8\rangle$}	{$\langle 0.3,0.5\rangle ,\langle 0.4,0.5\rangle$}
$Q_5$	{$\langle 0.1,0.4\rangle ,\langle 0.2,0.6\rangle$}	{$\langle 0.1,0.7\rangle ,\langle 0.2,0.3\rangle$}	{$\langle 0.6,0.2\rangle ,\langle 0.7,0.8\rangle$}	{$\langle 0.1,0.8\rangle ,\langle 0.2,0.2\rangle$}	{$\langle 0.2,0.3\rangle ,\langle 0.3,0.7\rangle$}	{$\langle 0.5,0.5\rangle ,\langle 0.6,0.5\rangle$}	{$\langle 0.6,0.6\rangle ,\langle 0.7,0.4\rangle$}	{$\langle 0.4,0.5\rangle ,\langle 0.5,0.5\rangle$}
$Q_6$	{$\langle 0.1,0.3\rangle ,\langle 0.2,0.7\rangle$}	{$\langle 0.3,0.4\rangle ,\langle 0.4,0.6\rangle$}	{$\langle 0.2,0.4\rangle ,\langle 0.3,0.6\rangle$}	{$\langle 0.2,0.5\rangle ,\langle 0.3,0.5\rangle$}	{$\langle 0.1,0.5\rangle ,\langle 0.2,0.5\rangle$}	{$\langle 0.7,0.6\rangle ,\langle 0.8,0.4\rangle$}	{$\langle 0.6,0.8\rangle ,\langle 0.7,0.2\rangle$}	{$\langle 0.3,0.7\rangle ,\langle 0.4,0.3\rangle$}

**Table 7:** The unified probabilistic hesitant fuzzy decision matrix.

	$P_1$	$P_2$	$P_3$	$P_4$
$Q_1$	{$\langle 0.2,0.2\rangle ,\langle 0.2,0.1\rangle ,\langle 0.2,0.\rangle ,\langle 0.2,0.1\rangle$,	{$\langle 0.1,0.2\rangle ,\langle 0.1,0.1\rangle ,\langle 0.1,0.1\rangle ,\langle 0.1,0.1\rangle$,	{$\langle 0.1,0.2\rangle ,\langle 0.1,0.1\rangle ,\langle 0.2,0.1\rangle ,\langle 0.2,0.1\rangle$,	{$\langle 0.2,0.2\rangle ,\langle 0.2,0.1\rangle ,\langle 0.2,0.1\rangle ,\langle 0.3,0.1\rangle$,
	$\langle 0.3,0.1\rangle ,\langle 0.3,0.1\rangle ,\langle 0.3,0.1\rangle ,\langle 0.3,0.2\rangle$}	$\langle 0.2,0.1\rangle ,\langle 0.2,0.1\rangle ,\langle 0.2,0.1\rangle ,\langle 0.2,0.2\rangle$}	$\langle 0.2,0.1\rangle ,\langle 0.2,0.1\rangle ,\langle 0.2,0.1\rangle ,\langle 0.2,0.2\rangle$}	$\langle 0.3,0.1\rangle ,\langle 0.3,0.1\rangle ,\langle 0.3,0.1\rangle ,\langle 0.3,0.2\rangle$}
$Q_2$	{$\langle 0.1,0.2\rangle ,\langle 0.1,0.1\rangle ,\langle 0.2,0.1\rangle ,\langle 0.2,0.1\rangle$,	{$\langle 0.2,0.2\rangle ,\langle 0.2,0.1\rangle ,\langle 0.2,0.1\rangle ,\langle 0.2,0.1\rangle$,	{$\langle 0.2,0.2\rangle ,\langle 0.3,0.1\rangle ,\langle 0.3,0.1\rangle ,\langle 0.3,0.1\rangle$,	{$\langle 0.1,0.2\rangle ,\langle 0.1,0.1\rangle ,\langle 0.1,0.1\rangle ,\langle 0.1,0.1\rangle$,
	$\langle 0.2,0.1\rangle ,\langle 0.2,0.1\rangle ,\langle 0.2,0.1\rangle ,\langle 0.2,0.2\rangle$}	$\langle 0.3,0.1\rangle ,\langle 0.3,0.1\rangle ,\langle 0.3,0.1\rangle ,\langle 0.3,0.2\rangle$}	$\langle 0.3,0.1\rangle ,\langle 0.3,0.1\rangle ,\langle 0.3,0.1\rangle ,\langle 0.3,0.2\rangle$}	$\langle 0.2,0.1\rangle ,\langle 0.2,0.1\rangle ,\langle 0.2,0.1\rangle ,\langle 0.2,0.2\rangle$}
$Q_3$	{$\langle 0.6,0.2\rangle ,\langle 0.6,0.1\rangle ,\langle 0.6,0.1\rangle ,\langle 0.6,0.1\rangle$,	{$\langle 0.7,0.2\rangle ,\langle 0.7,0.1\rangle ,\langle 0.7,0.1\rangle ,\langle 0.7,0.1\rangle$,	{$\langle 0.5,0.2\rangle ,\langle 0.5,0.1\rangle ,\langle 0.6,0.1\rangle ,\langle 0.6,0.1\rangle$,	{$\langle 0.6,0.2\rangle ,\langle 0.6,0.1\rangle ,\langle 0.6,0.1\rangle ,\langle 0.7,0.1\rangle ,$
	$\langle 0.7,0.1\rangle ,\langle 0.7,0.1\rangle ,\langle 0.7,0.1\rangle ,\langle 0.7,0.2\rangle$}	$\langle 0.7,0.1\rangle ,\langle 0.7,0.1\rangle ,\langle 0.8,0.1\rangle ,\langle 0.8,0.2\rangle$}	$\langle 0.6,0.1\rangle ,\langle 0.6,0.1\rangle ,\langle 0.6,0.1\rangle ,\langle 0.6,0.2\rangle$}	$\langle 0.7,0.1\rangle ,\langle 0.7,0.1\rangle ,\langle 0.7,0.1\rangle ,\langle 0.7,0.2\rangle$}
$Q_4$	{$\langle 0.2,0.2\rangle ,\langle 0.2,0.1\rangle ,\langle 0.2,0.1\rangle ,\langle 0.2,0.1\rangle$,	{$\langle 0.2,0.2\rangle ,\langle 0.3,0.1\rangle ,\langle 0.3,0.1\rangle ,\langle 0.3,0.1\rangle$,	{$\langle 0.2,0.2\rangle ,\langle 0.2,0.1\rangle ,\langle 0.2,0.1\rangle ,\langle 0.2,0.1\rangle$,	{$\langle 0.2,0.2\rangle ,\langle 0.2,0.1\rangle ,\langle 0.2,0.1\rangle ,\langle 0.2,0.1\rangle$,
	$\langle 0.3,0.1\rangle ,\langle 0.3,0.1\rangle ,\langle 0.3,0.1\rangle ,\langle 0.3,0.2\rangle$}	$\langle 0.3,0.1\rangle ,\langle 0.3,0.1\rangle ,\langle 0.3,0.1\rangle ,\langle 0.3,0.2\rangle$}	$\langle 0.2,0.1\rangle ,\langle 0.3,0.1\rangle ,\langle 0.3,0.1\rangle ,\langle 0.3,0.2\rangle$}	$\langle 0.3,0.1\rangle ,\langle 0.3,0.1\rangle ,\langle 0.3,0.1\rangle ,\langle 0.3,0.2\rangle$}
$Q_5$	{$\langle 0.4,0.2\rangle ,\langle 0.4,0.\rangle ,\langle 0.5,0.1\rangle ,\langle 0.5,0.1\rangle$,	{$\langle 0.3,0.2\rangle ,\langle 0.3,0.1\rangle ,\langle 0.3,0.1\rangle ,\langle 0.4,0.1\rangle$,	{$\langle 0.3,0.2\rangle ,\langle 0.3,0.1\rangle ,\langle 0.3,0.1\rangle ,\langle 0.3,0.1\rangle$,	{$\langle 0.3,0.2\rangle ,\langle 0.3,0.1\rangle ,\langle 0.4,0.1\rangle ,\langle 0.4,0.1\rangle$,
	$\langle 0.5,0.1\rangle ,\langle 0.5,0.1\rangle ,\langle 0.5,0.1\rangle ,\langle 0.5,0.2\rangle$}	$\langle 0.4,0.1\rangle ,\langle 0.4,0.1\rangle ,\langle 0.4,0.1\rangle ,\langle 0.4,0.2\rangle$}	$\langle 0.3,0.1\rangle ,\langle 0.3,0.1\rangle ,\langle 0.4,0.1\rangle ,\langle 0.4,0.2\rangle$}	$\langle 0.4,0.1\rangle ,\langle 0.4,0.1\rangle ,\langle 0.4,0.1\rangle ,\langle 0.4,0.2\rangle$}
$Q_6$	{$\langle 0.5,0.2\rangle ,\langle 0.6,0.1\rangle ,\langle 0.6,0.1\rangle ,\langle 0.6,0.1\rangle$,	{$\langle 0.5,0.2\rangle ,\langle 0.5,0.1\rangle ,\langle 0.6,0.1\rangle ,\langle 0.6,0.1\rangle$,	{$\langle 0.6,0.2\rangle ,\langle 0.6,0.1\rangle ,\langle 0.6,0.1\rangle ,\langle 0.6,0.1\rangle$,	{$\langle 0.5,0.2\rangle ,\langle 0.6,0.1\rangle ,\langle 0.6,0.1\rangle ,\langle 0.6,0.1\rangle$,
	$\langle 0.6,0.1\rangle ,\langle 0.6,0.1\rangle ,\langle 0.6,0.1\rangle ,\langle 0.6,0.2\rangle$}	$\langle 0.6,0.1\rangle ,\langle 0.6,0.1\rangle ,\langle 0.6,0.1\rangle ,\langle 0.6,0.2\rangle$}	$\langle 0.7,0.1\rangle ,\langle 0.7,0.1\rangle ,\langle 0.7,0.1\rangle ,\langle 0.7,0.2\rangle$}	$\langle 0.6,0.1\rangle ,\langle 0.6,0.1\rangle ,\langle 0.6,0.1\rangle ,\langle 0.6,0.2\rangle$}

	$P_5$	$P_6$	$P_7$	$P_8$
$Q_1$	{$\langle 0.7,0.2\rangle ,\langle 0.7,0.1\rangle ,\langle 0.7,0.1\rangle ,\langle 0.7,0.1\rangle$,	{$\langle 0.1,0.2\rangle ,\langle 0.1,0.1\rangle ,\langle 0.1,0.1\rangle ,\langle 0.1,0.1\rangle$,	{$\langle 0.4,0.2\rangle ,\langle 0.4,0.1\rangle ,\langle 0.4,0.1\rangle ,\langle 0.5,0.1\rangle$,	{$\langle 0.4,0.2\rangle ,\langle 0.4,0.1\rangle ,\langle 0.5,0.1\rangle ,\langle 0.5,0.1\rangle$,
	$\langle 0.8,0.1\rangle ,\langle 0.8,0.1\rangle ,\langle 0.8,0.1\rangle ,\langle 0.8,0.2\rangle$}	$\langle 0.2,0.1\rangle ,\langle 0.2,0.1\rangle ,\langle 0.2,0.1\rangle ,\langle 0.2,0.2\rangle$}	$\langle 0.5,0.1\rangle ,\langle 0.5,0.1\rangle ,\langle 0.5,0.1\rangle ,\langle 0.5,0.2\rangle$}	$\langle 0.5,0.1\rangle ,\langle 0.5,0.1\rangle ,\langle 0.5,0.1\rangle ,\langle 0.5,0.2\rangle$}
$Q_2$	{$\langle 0.8,0.2\rangle ,\langle 0.8,0.1\rangle ,\langle 0.9,0.1\rangle ,\langle 0.9,0.1\rangle$,	{$\langle 0.3,0.2\rangle ,\langle 0.3,0.1\rangle ,\langle 0.4,0.1\rangle ,\langle 0.4,0.1\rangle$,	{$\langle 0.5,0.2\rangle ,\langle 0.5,0.1\rangle ,\langle 0.6,0.1\rangle ,\langle 0.6,0.1\rangle$,	{$\langle 0.6,0.2\rangle ,\langle 0.6,0.1\rangle ,\langle 0.6,0.1\rangle ,\langle 0.6,0.1\rangle$,
	$\langle 0.9,0.1\rangle ,\langle 0.9,0.1\rangle ,\langle 0.9,0.1\rangle ,\langle 0.9,0.2\rangle$}	$\langle 0.4,0.1\rangle ,\langle 0.4,0.1\rangle ,\langle 0.4,0.1\rangle ,\langle 0.4,0.2\rangle$}	$\langle 0.6,0.1\rangle ,\langle 0.6,0.1\rangle ,\langle 0.6,0.1\rangle ,\langle 0.6,0.2\rangle$}	$\langle 0.7,0.1\rangle ,\langle 0.7,0.1\rangle ,\langle 0.7,0.1\rangle ,\langle 0.7,0.2\rangle$}
$Q_3$	{$\langle 0.1,0.2\rangle ,\langle 0.1,0.1\rangle ,\langle 0.1,0.1\rangle ,\langle 0.2,0.1\rangle$,	{$\langle 0.3,0.2\rangle ,\langle 0.3,0.1\rangle ,\langle 0.3,0.1\rangle ,\langle 0.3,0.1\rangle$,	{$\langle 0.1,0.2\rangle ,\langle 0.1,0.1\rangle ,\langle 0.1,0.1\rangle ,\langle 0.1,0.1\rangle$,	{$\langle 0.1,0.2\rangle ,\langle 0.1,0.1\rangle ,\langle 0.1,0.1\rangle ,\langle 0.2,0.1\rangle$,
	$\langle 0.2,0.1\rangle ,\langle 0.2,0.1\rangle ,\langle 0.2,0.1\rangle ,\langle 0.2,0.2\rangle$}	$\langle 0.3,0.1\rangle ,\langle 0.3,0.1\rangle ,\langle 0.3,0.1\rangle ,\langle 0.4,0.2\rangle$}	$\langle 0.2,0.1\rangle ,\langle 0.2,0.1\rangle ,\langle 0.2,0.1\rangle ,\langle 0.2,0.2\rangle$}	$\langle 0.2,0.1\rangle ,\langle 0.2,0.1\rangle ,\langle 0.2,0.1\rangle ,\langle 0.2,0.2\rangle$}
$Q_4$	{$\langle 0.6,0.2\rangle ,\langle 0.7,0.1\rangle ,\langle 0.7,0.1\rangle ,\langle 0.7,0.1\rangle$,	{$\langle 0.7,0.2\rangle ,\langle 0.7,0.1\rangle ,\langle 0.8,0.1\rangle ,\langle 0.8,0.1\rangle$,	{$\langle 0.3,0.2\rangle ,\langle 0.3,0.1\rangle ,\langle 0.3,0.1\rangle ,\langle 0.3,0.1\rangle$,	{$\langle 0.4,0.2\rangle ,\langle 0.4,0.1\rangle ,\langle 0.4,0.1\rangle ,\langle 0.4,0.1\rangle$,
	$\langle 0.7,0.1\rangle ,\langle 0.7,0.1\rangle ,\langle 0.7,0.1\rangle ,\langle 0.7,0.2\rangle$}	$\langle 0.8,0.1\rangle ,\langle 0.8,0.1\rangle ,\langle 0.8,0.1\rangle ,\langle 0.8,0.2\rangle$}	$\langle 0.3,0.1\rangle ,\langle 0.3,0.1\rangle ,\langle 0.3,0.1\rangle ,\langle 0.4,0.2\rangle$}	$\langle 0.4,0.1\rangle ,\langle 0.4,0.1\rangle ,\langle 0.5,0.1\rangle ,\langle 0.5,0.2\rangle$}
$Q_5$	{$\langle 0.4,0.2\rangle ,\langle 0.4,0.1\rangle ,\langle 0.4,0.1\rangle ,\langle 0.4,0.1\rangle$,	{$\langle 0.6,0.2\rangle ,\langle 0.6,0.1\rangle ,\langle 0.6,0.1\rangle ,\langle 0.7,0.1\rangle ,$	{$\langle 0.2,0.2\rangle ,\langle 0.2,0.1\rangle ,\langle 0.2,0.1\rangle ,\langle 0.2,0.1\rangle$,	{$\langle 0.5,0.2\rangle ,\langle 0.5,0.1\rangle ,\langle 0.5,0.1\rangle ,\langle 0.6,0.1\rangle$,
	$\langle 0.5,0.1\rangle ,\langle 0.5,0.1\rangle ,\langle 0.5,0.1\rangle ,\langle 0.5,0.2\rangle$}	$\langle 0.7,0.1\rangle ,\langle 0.7,0.1\rangle ,\langle 0.7,0.1\rangle ,\langle 0.7,0.2\rangle$}	$\langle 0.3,0.1\rangle ,\langle 0.3,0.1\rangle ,\langle 0.3,0.1\rangle ,\langle 0.3,0.2\rangle$}	$\langle 0.6,0.1\rangle ,\langle 0.6,0.1\rangle ,\langle 0.6,0.1\rangle ,\langle 0.6,0.2\rangle$}
$Q_6$	{$\langle 0.3,0.2\rangle ,\langle 0.3,0.1\rangle ,\langle 0.3,0.1\rangle ,\langle 0.3,0.1\rangle$,	{$\langle 0.5,0.2\rangle ,\langle 0.5,0.1\rangle ,\langle 0.5,0.1\rangle ,\langle 0.5,0.1\rangle$,	{$\langle 0.2,0.2\rangle ,\langle 0.2,0.1\rangle ,\langle 0.2,0.1\rangle ,\langle 0.2,0.1\rangle$,	{$\langle 0.4,0.2\rangle ,\langle 0.4,0.1\rangle ,\langle 0.4,0.1\rangle ,\langle 0.4,0.1\rangle$,
	$\langle 0.3,0.1\rangle ,\langle .4,0.1\rangle ,\langle 0.4,0.1\rangle ,\langle 0.4,0.2\rangle$}	$\langle 0.7,0.1\rangle ,\langle 0.7,0.1\rangle ,\langle 0.7,0.1\rangle ,\langle 0.7,0.2\rangle$}	$\langle 0.2,0.1\rangle ,\langle 0.3,0.1\rangle ,\langle 0.3,0.1\rangle ,\langle 0.3,0.2\rangle$}	$\langle 0.4,0.1\rangle ,\langle 0.4,0.1\rangle ,\langle 0.4,0.1\rangle ,\langle 0.5,0.2\rangle$}

	$P_9$	$P_{10}$	$P_{11}$	$P_{12}$
$Q_1$	{$\langle 0.1,0.2\rangle ,\langle 0.1,0.1\rangle ,\langle 0.1,0.1\rangle ,\langle 0.1,0.1\rangle$,	{$\langle 0.7,0.2\rangle ,\langle 0.8,0.1\rangle ,\langle 0.8,0.1\rangle ,\langle 0.8,0.1\rangle$,	{$\langle 0.2,0.2\rangle ,\langle 0.2,0.1\rangle ,\langle 0.3,0.1\rangle ,\langle 0.3,0.1\rangle$,	{$\langle 0.3,0.2\rangle ,\langle 0.3,0.1\rangle ,\langle 0.3,0.1\rangle ,\langle 0.4,0.1\rangle$,
	$\langle 0.2,0.1\rangle ,\langle 0.2,0.1\rangle ,\langle 0.2,0.1\rangle ,\langle 0.2,0.2\rangle$}	$\langle 0.8,0.1\rangle ,\langle 0.8,0.1,\rangle ,\langle ,0.8,0.1\rangle ,\langle 0.8,0.2\rangle$}	$\langle 0.3,0.1\rangle ,\langle 0.3,0.1\rangle ,\langle 0.3,0.1\rangle ,\langle 0.3,0.2\rangle$}	$\langle 0.4,0.1\rangle ,\langle 0.4,0.1\rangle ,\langle 0.4,0.1\rangle ,\langle 0.4,0.2\rangle$}
$Q_2$	{$\langle 0.5,0.2\rangle ,\langle 0.6,0.1\rangle ,\langle 0.6,0.1\rangle ,\langle 0.6,0.1\rangle$,	{$\langle 0.8,0.2\rangle ,\langle 0.8,0.1\rangle ,\langle 0.8,0.1\rangle ,\langle 0.8,0.1\rangle$,	{$\langle 0.1,0.2\rangle ,\langle 0.2,0.1\rangle ,\langle 0.2,0.1\rangle ,\langle 0.2,0.1\rangle$,	{$\langle 0.1,0.2\rangle ,\langle 0.1,0.1\rangle ,\langle 0.2,0.1\rangle ,\langle 0.2,0.1\rangle$,
	$\langle 0.6,0.1\rangle ,\langle 0.6,0.1\rangle ,\langle 0.6,0.1\rangle ,\langle 0.6,0.2\rangle$}	$\langle 0.8,0.1\rangle ,\langle 0.8,0.1\rangle ,\langle 0.8,0.1\rangle ,\langle 0.9,0.2\rangle$}	$\langle 0.2,0.1\rangle ,\langle 0.2,0.1\rangle ,\langle 0.2,0.1\rangle ,\langle 0.2,0.2\rangle$}	$\langle 0.2,0.1\rangle ,\langle 0.2,0.1\rangle ,\langle 0.2,0.1\rangle ,\langle 0.2,0.2\rangle$}
$Q_3$	{$\langle 0.3,0.2\rangle ,\langle 0.3,0.1\rangle ,\langle 0.3,0.1\rangle ,\langle 0.3,0.1\rangle$,	{$\langle 0.2,0.2\rangle ,\langle 0.3,0.1\rangle ,\langle 0.3,0.1\rangle ,\langle 0.3,0.1\rangle$,	{$\langle 0.4,0.2\rangle ,\langle 0.4,0.1\rangle ,\langle 0.4,0.1\rangle ,\langle 0.5,0.1\rangle$,	{$\langle 0.2,0.2\rangle ,\langle 0.2,0.1\rangle ,\langle 0.2,0.1\rangle ,\langle 0.2,0.1\rangle$,
	$\langle 0.3,0.1\rangle ,\langle 0.3,0.1\rangle ,\langle 0.3,0.1\rangle ,\langle 0.4,0.2\rangle$}	$\langle 0.3,0.1\rangle ,\langle 0.3,0.1\rangle ,\langle 0.3,0.1\rangle ,\langle 0.3,0.2\rangle$}	$\langle 0.5,0.1\rangle ,\langle 0.4,0.1\rangle ,\langle 0.5,0.1\rangle ,\langle 0.5,0.2\rangle$}	$\langle 0.2,0.1\rangle ,\langle 0.3,0.1\rangle ,\langle 0.3,0.1\rangle ,\langle 0.3,0.2\rangle$}
$Q_4$	{$\langle 0.2,0.2\rangle ,\langle 0.2,0.1\rangle ,\langle 0.2,0.1\rangle ,\langle 0.2,0.1\rangle$,	{$\langle 0.2,0.2\rangle ,\langle 0.2,0.1\rangle ,\langle 0.3,0.1\rangle ,\langle 0.3,0.1\rangle$,	{$\langle 0.3,0.2\rangle ,\langle 0.3,0.1\rangle ,\langle 0.3,0.1\rangle ,\langle 0.4,0.1\rangle$,	{$\langle 0.3,0.2\rangle ,\langle 0.3,0.1\rangle ,\langle 0.3,0.1\rangle ,\langle 0.4,0.1\rangle$,
	$\langle 0.2,0.1\rangle ,\langle 0.3,0.1\rangle ,\langle 0.3,0.1\rangle ,\langle 0.3,0.2\rangle$}	$\langle 0.3,0.1\rangle ,\langle 0.3,0.1\rangle ,\langle 0.3,0.1\rangle ,\langle 0.3,0.2\rangle$}	$\langle 0.4,0.1\rangle ,\langle 0.4,0.1\rangle ,\langle 0.4,0.1\rangle ,\langle 0.4,0.2\rangle$}	$\langle 0.4,0.1\rangle ,\langle 0.4,0.1\rangle ,\langle 0.4,0.1\rangle ,\langle 0.4,0.2\rangle$}
$Q_5$	{$\langle 0.1,0.2\rangle ,\langle 0.1,0.1\rangle ,\langle 0.1,0.1\rangle ,\langle 0.2,0.1\rangle$,	{$\langle 0.1,0.2\rangle ,\langle 0.1,0.1\rangle ,\langle 0.1,0.1\rangle ,\langle 0.1,0.1\rangle$,	{$\langle 0.6,0.2\rangle ,\langle 0.7,0.1\rangle ,\langle 0.7,0.1\rangle ,\langle 0.7,0.1\rangle$,	{$\langle 0.1,0.2\rangle ,\langle 0.1,0.1\rangle ,\langle 0.1,0.1\rangle ,\langle 0.1,0.1\rangle$,
	$\langle 0.2,0.1\rangle ,\langle 0.2,0.1\rangle ,\langle 0.2,0.1\rangle ,\langle 0.2,0.2\rangle$}	$\langle 0.1,0.1\rangle ,\langle 0.1,0.1\rangle ,\langle 0.2,0.1\rangle ,\langle 0.2,0.2\rangle$}	$\langle 0.7,0.1\rangle ,\langle 0.7,0.1\rangle ,\langle 0.7,0.1\rangle ,\langle 0.7,0.2\rangle$}	$\langle 0.1,0.1\rangle ,\langle 0.1,0.1\rangle ,\langle 0.1,0.1\rangle ,\langle 0.2,0.2\rangle$}
$Q_6$	{$\langle 0.1,0.2\rangle ,\langle 0.1,0.1\rangle ,\langle 0.2,0.1\rangle ,\langle 0.2,0.1\rangle$,	{$\langle 0.3,0.2\rangle ,\langle 0.3,0.1\rangle ,\langle 0.3,0.1\rangle ,\langle 0.4,0.1\rangle$,	{$\langle 0.2,0.2\rangle ,\langle 0.2,0.1\rangle ,\langle 0.2,0.1\rangle ,\langle 0.3,0.1\rangle$,	{$\langle 0.2,0.2\rangle ,\langle 0.2,0.1\rangle ,\langle 0.2,0.1\rangle ,\langle 0.2,0.1\rangle$,
	$\langle 0.2,0.1\rangle ,\langle 0.2,0.1\rangle ,\langle 0.2,0.1\rangle ,\langle 0.2,0.2\rangle$}	$\langle 0.4,0.1\rangle ,\langle 0.4,0.1\rangle ,\langle 0.4,0.1\rangle ,\langle 0.4,0.2\rangle$}	$\langle 0.3,0.1\rangle ,\langle 0.3,0.1\rangle ,\langle 0.3,0.1\rangle ,\langle 0.3,0.2\rangle$}	$\langle 0.3,0.1\rangle ,\langle 0.3,0.1\rangle ,\langle 0.3,0.1\rangle ,\langle 0.3,0.2\rangle$}

	$P_{13}$	$P_{14}$	$P_{15}$	$P_{16}$
$Q_1$	{$\langle 0.7,0.2\rangle ,\langle 0.7,0.1\rangle ,\langle 0.7,0.1\rangle ,\langle 0.7,0.1\rangle$,	{$\langle 0.6,0.2\rangle ,\langle 0.6,0.1\rangle ,\langle 0.6,0.1\rangle ,\langle 0.6,0.1\rangle$,	{$\langle 0.7,0.2\rangle ,\langle 0.7,0.1\rangle ,\langle 0.7,0.1\rangle ,\langle 0.7,0.1\rangle$,	{$\langle 0.4,0.2\rangle ,\langle 0.5,0.1\rangle ,\langle 0.5,0.1\rangle ,\langle 0.5,0.1\rangle$,
	$\langle 0.8,0.1\rangle ,\langle 0.8,0.1\rangle ,\langle 0.8,0.1\rangle ,\langle 0.8,0.2\rangle$}	$\langle 0.7,0.1\rangle ,\langle 0.7,0.1\rangle ,\langle 0.7,0.1\rangle ,\langle 0.7,0.2\rangle$}	$\langle 0.7,0.1\rangle ,\langle 0.7,0.1\rangle ,\langle 0.8,0.1\rangle ,\langle 0.8,0.2\rangle$}	$\langle 0.5,0.1\rangle ,\langle 0.5,0.1\rangle ,\langle 0.5,0.1\rangle ,\langle 0.5,0.2\rangle$}
$Q_2$	{$\langle 0.8,0.2\rangle ,\langle 0.8,0.1\rangle ,\langle 0.9,0.1\rangle ,\langle 0.9,0.1\rangle$,	{$\langle 0.7,0.2\rangle ,\langle 0.8,0.1\rangle ,\langle 0.8,0.1\rangle ,\langle 0.8,0.1\rangle$,	{$\langle 0.8,0.2\rangle ,\langle 0.8,0.1\rangle ,\langle 0.9,0.1\rangle ,\langle 0.9,0.1\rangle$,	{$\langle 0.6,0.2\rangle ,\langle 0.6,0.1\rangle ,\langle 0.6,0.1\rangle ,\langle 0.7,0.1\rangle$,
	$\langle 0.9,0.1\rangle ,\langle 0.9,0.1\rangle ,\langle 0.9,0.1\rangle ,\langle 0.9,0.2\rangle$}	$\langle 0.8,0.1\rangle ,\langle 0.8,0.1\rangle ,\langle 0.8,0.1\rangle ,\langle 0.8,0.2\rangle$}	$\langle 0.9,0.1\rangle ,\langle 0.9,0.1\rangle ,\langle 0.9,0.1\rangle ,\langle 0.9,0.2\rangle$}	$\langle 0.7,0.1\rangle ,\langle 0.7,0.1\rangle ,\langle 0.7,0.1\rangle ,\langle 0.7,0.2\rangle$}
$Q_3$	{$\langle 0.1,0.2\rangle ,\langle 0.1,0.1\rangle ,\langle 0.1,0.1\rangle ,\langle 0.1,0.1\rangle$,	{$\langle 0.1,0.2\rangle ,\langle 0.1,0.1\rangle ,\langle 0.1,0.1\rangle ,\langle 0.1,0.1\rangle$,	{$\langle 0.4,0.2\rangle ,\langle 0.4,0.1\rangle ,\langle 0.4,0.1\rangle ,\langle 0.4,0.1\rangle$,	{$\langle 0.2,0.2\rangle ,\langle 0.2,0.1\rangle ,\langle 0.3,0.1\rangle ,\langle 0.3,0.1\rangle$,
	$\langle 0.2,0.1\rangle ,\langle 0.2,0.1\rangle ,\langle 0.2,0.1\rangle ,\langle 0.2,0.2\rangle$}	$\langle 0.1,0.1\rangle ,\langle 0.2,0.1\rangle ,\langle 0.2,0.1\rangle ,\langle 0.2,0.2\rangle$}	$\langle 0.5,0.1\rangle ,\langle 0.5,0.1\rangle ,\langle 0.5,0.1\rangle ,\langle 0.5,0.2\rangle$}	$\langle 0.3,0.1\rangle ,\langle 0.3,0.1\rangle ,\langle 0.3,0.1\rangle ,\langle 0.3,0.2\rangle$}
$Q_4$	{$\langle 0.5,0.2\rangle ,\langle 0.5,0.1\rangle ,\langle 0.5,0.1\rangle ,\langle 0.5,0.1\rangle$,	{$\langle 0.7,0.2\rangle ,\langle 0.8,0.1\rangle ,\langle 0.8,0.1\rangle ,\langle 0.8,0.1\rangle$,	{$\langle 0.7,0.2\rangle ,\langle 0.8,0.1\rangle ,\langle 0.8,0.1\rangle ,\langle 0.8,0.1\rangle$,	{$\langle 0.4,0.2\rangle ,\langle 0.4,0.1\rangle ,\langle 0.4,0.1\rangle ,\langle 0.4,0.1\rangle$,
	$\langle 0.5,0.1\rangle ,\langle 0.5,0.1\rangle ,\langle 0.6,0.1\rangle ,\langle 0.6,0.2\rangle$}	$\langle 0.8,0.1\rangle ,\langle 0.8,0.1\rangle ,\langle 0.8,0.1\rangle ,\langle 0.8,0.2\rangle$}	$\langle 0.8,0.1\rangle ,\langle 0.8,0.1\rangle ,\langle 0.8,0.1\rangle ,\langle 0.8,0.2\rangle$}	$\langle 0.5,0.1\rangle ,\langle 0.5,0.1\rangle ,\langle 0.5,0.1\rangle ,\langle 0.5,0.2\rangle$}
$Q_5$	{$\langle 0.2,0.2\rangle ,\langle 0.2,0.1\rangle ,\langle 0.3,0.1\rangle ,\langle 0.3,0.1\rangle$,	{$\langle 0.5,0.2\rangle ,\langle 0.5,0.1\rangle ,\langle 0.5,0.1\rangle ,\langle 0.5,0.1\rangle$,	{$\langle 0.6,0.2\rangle ,\langle 0.6,0.1\rangle ,\langle 0.6,0.1\rangle ,\langle 0.6,0.1\rangle$,	{$\langle 0.3,0.2\rangle ,\langle 0.3,0.1\rangle ,\langle 0.3,0.1\rangle ,\langle 0.3,0.1\rangle$,
	$\langle 0.3,0.1\rangle ,\langle 0.3,0.1\rangle ,\langle 0.3,0.1\rangle ,\langle 0.3,0.2\rangle$}	$\langle 0.6,0.1\rangle ,\langle 0.6,0.1\rangle ,\langle 0.6,0.1\rangle ,\langle 0.6,0.2\rangle$}	$\langle 0.6,0.1\rangle ,\langle 0.7,0.1\rangle ,\langle 0.7,0.1\rangle ,\langle 0.7,0.2\rangle$}	$\langle 0.4,0.1\rangle ,\langle 0.4,0.1\rangle ,\langle 0.4,0.1\rangle ,\langle 0.4,0.2\rangle$}
$Q_6$	{$\langle 0.1,0.2\rangle ,\langle 0.1,0.1\rangle ,\langle 0.1,0.1\rangle ,\langle 0.1,0.1\rangle$,	{$\langle 0.1,0.2\rangle ,\langle 0.1,0.1\rangle ,\langle 0.2,0.1\rangle ,\langle 0.2,0.1\rangle$,	{$\langle 0.6,0.2\rangle ,\langle 0.6,0.1\rangle ,\langle 0.6,0.1\rangle ,\langle 0.6,0.1\rangle$	{$\langle 0.3,0.2\rangle ,\langle 0.3,0.1\rangle ,\langle 0.3,0.1\rangle ,\langle 0.3,0.1\rangle$,
	$\langle 0.2,0.1\rangle ,\langle 0.2,0.1\rangle ,\langle 0.2,0.1\rangle ,\langle 0.2,0.2\rangle$}	$\langle 0.2,0.1\rangle ,\langle 0.2,0.1\rangle ,\langle 0.2,0.1\rangle ,\langle 0.2,0.2\rangle$}	$\langle 0.6,0.1\rangle ,\langle 0.6,0.1\rangle ,\langle 0.6,0.1\rangle ,\langle 0.7,0.2\rangle$}	$\langle 0.3,0.1\rangle ,\langle 0.3,0.1\rangle ,\langle 0.4,0.1\rangle ,\langle 0.4,0.2\rangle$}

**Table 8:** The normalized unified probabilistic hesitant fuzzy decision matrix (NUPHFDM).

	$P_1$	$P_2$	$P_3$	$P_4$	$P_5$	$P_6$	$P_7$	$P_8$	$P_9$	$P_{10}$	$P_{11}$	$P_{12}$	$P_{13}$	$P_{14}$	$P_{15}$	$P_{16}$
$Q_1$	0.68333	1	1	0.56667	0.882	0.1929	0.8067	0.7238	1	0.1667	0.666667	0.4667	0.8624	0.8339	0.8403	0.7286
$Q_2$	1	0.58333	0.6	1	1	0.4786	1	1	0.2567	0.1569	1	1	1	1	1	1
$Q_3$	0.25952	0.203571	0.29333	0.22381	0.211	0.4661	0.26	0.2429	0.4667	0.3467	0.39	0.7167	0.1707	0.1786	0.5167	0.4071
$Q_4$	0.68333	0.53333	0.71776	0.56667	0.764	1	0.56	0.6619	0.6133	0.4833	0.4467	0.4667	0.6381	0.95	0.8972	0.6809
$Q_5$	0.355	0.40833	0.51667	0.4	0.5529	0.8571	0.4767	0.8619	0.95	1	0.2905	1	0.3081	0.7054	0.7361	0.5286
$Q_6$	0.29	0.26	0.259524	0.256667	0.4221	0.7143	0.4033	0.6476	0.9	0.85	0.7	0.6833	0.2054	0.2161	0.7139	0.5

**Table 9:** Weighted sum NUPHFDM.

	$P_1$	$P_2$	$P_3$	$P_4$	$P_5$	$P_6$	$P_7$	$P_8$	$P_9$	$P_{10}$	$P_{11}$	$P_{12}$	$P_{13}$	$P_{14}$	$P_{15}$	$P_{16}$
$Q_1$	0.265679	0.1579	0.0756	0.026577	0.071354	0.00544	0.012262	0.006659	0.0695	0.004634	0.0116	0.003874	0.037601	0.013759	0.006722	0.004372
$Q_2$	0.3888	0.092108	0.04536	0.0469	0.0809	0.013497	0.0152	0.0092	0.017841	0.004362	0.0174	0.0083	0.0436	0.0165	0.008	0.006
$Q_3$	0.100901	0.032144	0.022176	0.010497	0.01707	0.013144	0.003952	0.002235	0.032436	0.009638	0.006786	0.005949	0.007443	0.002947	0.004134	0.002443
$Q_4$	0.265679	0.084213	0.054263	0.026577	0.061808	0.0282	0.008512	0.006089	0.042624	0.013436	0.007773	0.003874	0.027821	0.015675	0.007178	0.004085
$Q_5$	0.138024	0.064475	0.03906	0.01876	0.04473	0.02417	0.007246	0.007929	0.066025	0.0278	0.005055	0.0083	0.013433	0.011639	0.005889	0.003172
$Q_6$	0.112752	0.041054	0.01962	0.012038	0.034148	0.020143	0.00613	0.005958	0.06255	0.02363	0.01218	0.005671	0.008955	0.003566	0.005711	0.003

**Table 10:** Weighted product NUPHFDM.

	$P_1$	$P_2$	$P_3$	$P_4$	$P_5$	$P_6$	$P_7$	$P_8$
$Q_1$	0.862391	1	1	0.973713	0.989893	0.954655	0.99674	0.997031
$Q_2$	1	0.918413	0.962118	1	1	0.979434	1	1
$Q_3$	0.591874	0.777761	0.911449	0.932201	0.881728	0.978703	0.979733	0.987065
$Q_4$	0.862391	0.905509	0.975241	0.973713	0.978458	1	0.991225	0.996211
$Q_5$	0.668542	0.868118	0.951303	0.957936	0.953191	0.995661	0.988802	0.998634
$Q_6$	0.617988	0.808396	0.90305	0.938209	0.932601	0.990557	0.986292	0.996011

	$P_9$	$P_{10}$	$P_{11}$	$P_{12}$	$P_{13}$	$P_{14}$	$P_{15}$	$P_{16}$
$Q_1$	1	0.951415	0.99297	0.993695	0.993566	0.997007	0.998609	0.998102
$Q_2$	0.909819	0.949813	1	1	1	1	1	1
$Q_3$	0.948414	0.970981	0.983749	0.997239	0.925817	0.971977	0.994732	0.994622
$Q_4$	0.966592	0.979989	0.986076	0.993695	0.980603	0.999154	0.999133	0.997697
$Q_5$	0.996441	1	0.978721	1	0.949964	0.994258	0.997552	0.996182
$Q_6$	0.992704	0.995492	0.993813	0.996844	0.933317	0.975039	0.997308	0.99585

## Result validation

### Comparison analysis

In this section, we compare the proposed method to the PHFEs-MCDM processes for AHP weights, which yield six alternative rankings that are perfectly comparable to the pattern of existing PHFEs-MCDM techniques.

**Figure 11:**
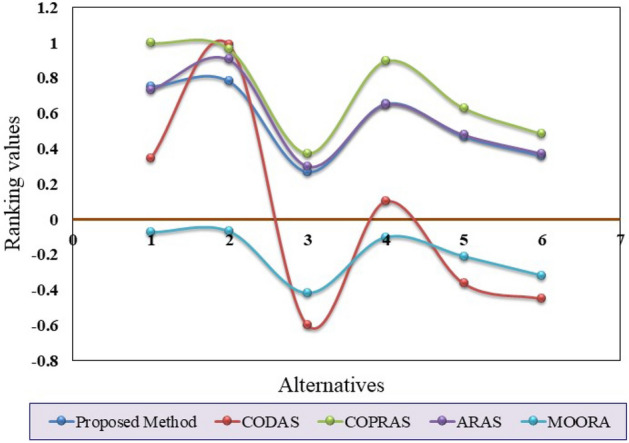
Comparison of ranking results.

Furthermore, when trying to compare WASPAS to the other four methods (CODAS, COPRAS, ARAS, and MOORA), the scoring results show slight changes. The purpose of the 0.01 level in the COPRAS method ($P_1$ is preferred over $P_2$) is that in MCDM, solution criteria weights contribute. The profitability criterion, which is used to calculate the COPRAS method, emphasises both beneficial and unfavourable criteria. The best choice in CODAS, ARAS, and MOORA is determined by how close the solution is to the ideal. The values are graphically depicted in Fig. 11 and recorded in Table 11. The ranking order of the extant MCDM is as follows:

CODAS : $$Q_{2}> Q_{1}> Q_{4}> Q_{5}> Q_{6} > Q_{3}$$

COPRAS : $$Q_{1}> Q_{2}> Q_{4}> Q_{5}> Q_{6} > Q_{3}$$

ARAS : $$Q_{2}> Q_{1}> Q_{4}> Q_{5}> Q_{6} > Q_{3}$$

MOORA : $$Q_{2}> Q_{1}> Q_{4}> Q_{5}> Q_{6} > Q_{3}$$

Although the PHFEs-WASPAS takes a little longer to return the result, it takes into account the competing criteria to prevent inconsistent and ambiguous decision-making. As a result, our suggested proposed method might be more practical and realistic in real-world MCDM applications.

**Table 11:** Comparison of ranking results.

Alternative	WASPAS	CODAS	COPRAS	ARAS	MOORA	Optimal rank
$Q_1$	0.75224	0.347035	1	0.73341	− 0.07425	2
$Q_2$	0.78093	0.988125	0.963834	0.90386	− 0.06894	1
$Q_3$	0.26822	-0.59957	0.37117	0.30074	− 0.41926	6
$Q_4$	0.65365	0.101017	0.89572	0.64986	− 0.10226	3
$Q_5$	0.4697	− 0.36249	0.628649	0.47833	− 0.21072	4
$Q_6$	0.35849	− 0.44948	0.482513	0.37232	− 0.3195	5

### Sensitivity analysis

The influence levels of impartial sub-criteria weights are investigated using the sensitivity analysis section. The main goal of the sensitivity report is to assess the priority ranking when objective weights are changed in significance. We have explained what changes occur in the sequence of alternatives when changing the relevance of selected sub-criteria, and this has been demonstrated by sensitivity analysis.

The sensitivity analysis is demonstrated in our scientific report using the two cases below.

*Case 1*:

When it comes to assigning weights, both desirable and undesirable sub-criteria are available prior to 0.5 for beneficial sub-criteria and 0.5 for negative pole. The DM in this event determines the best plastic bags. In this event, both equal impact and weight values are obtained. The weights of each sub-criteria are suspected to be $w_j$ =0.0625 (j = 1,2,..., 16) at each of the criteria $p_j$. Our recommended computational methods and the results shown in Table 12.

*Case 2*

**Figure 12:**
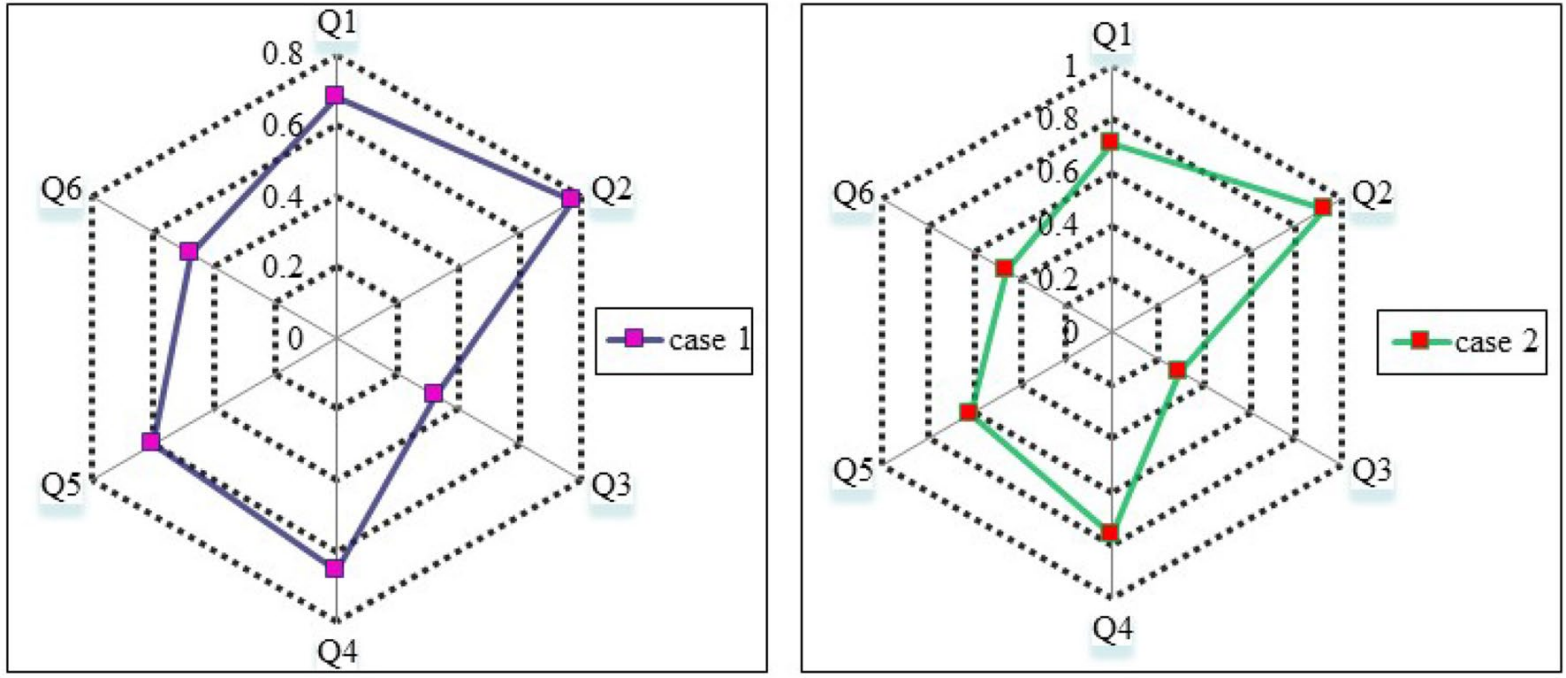
Sensitivity analysis results.

We provide equal weights for beneficial and the rest are assigned zero using the developed methodology the alternative $Q_2$ is obtained as the optimal solution. The obtained results for two cases are presented in Table 12 and Fig. 12. The proposed AHP weighting method detects weights for the sub-criteria with a focus on positive criteria in the segment. The PHFEs-WASPAS method is then used to choose one of the finest bio-plastic bags.

When it comes to the weight detection method, the AHP process is suitable when it appears differently in relation to the criteria. As a result, in terms of efficiency, the AHP-WASPAS using some methods outperforms the entropy process in the MCDM problem. When we contrast the ranking of proposed PHFEs with WASPAS, we find that the effects are in the same order.

**Table 12:** Sensitivity results.

	Ranking values	Ranking order
Case 1	0.6789, 0.7744, 0.3208, 0.6571, 0.5975, 0.4727	$Q_2> Q_1> Q_4> Q_5> Q_6 > Q_3$
Case 2	0.7050, 0.9234, 0.2942, 0.7616, 0.6134, 0.4556	$Q_2> Q_4> Q_1> Q_5> Q_6 > Q_3$
Proposed method	0.7522, 0.7809, 0.2682, 0.6536, 0.4696, 0.3584	$Q_2> Q_1> Q_4> Q_5 > Q_6$> $Q_3$
(AHP-WASPAS)		

## Conclusion

While most existing unification procedures for PHFEs suffer from various limitations, unifying logically the underling parameters is a critical step in decision making under uncertainty. As a result, we propose a new PHFE unification methodology that is simple and easy to understand, as opposed to the previous ones, which are intricate and convoluted. We also offered an attempt to improve the computation of division and subtraction operations on the UPHFEs. The unification procedure was then applied to AHP-WASPAS concerns to improve decision making, effectiveness, and efficiency. We chose the PHFS-WASPAS as the most effective fuzzy environment for bio-plastic bags as a consequence. The research study’s ranking results for the suggested PHFS-MCDM procedures outperformed the already-used AHP techniques by offering the best answer. The solution is to reduce the negative impact on the environment. For all environmental indicators with the exception of persistence, the reusable plastic bags (HDPE 70-μm, polyester woven, and polyester spun-woven) had significantly lower impacts than any of the single-use bag options. The number of times that bags are reused is a significant influence on the overall environmental impacts. Reusable bags have a smaller environmental impact than single-use plastic bags because they only need to be used 3–10 times. Although increasing the recycled content of the bioplastic bags significantly reduced the overall environmental consequences by up to 52%, the single-use 24-μm, long polyethylene bag with no recycled material had the highest environmental impacts. The high environmental impacts of plastic bags, whether compared to worldwide or European plastics manufacturing, are mostly attributable to the energy- and raw material-intensive coal-to-liquid polymer production process. Paper and imported polymers are now more appealing from an environmental standpoint. However, if particular circumstances demand the use of single-use bags, the bags should have a high recycled content and/or be composed of materials that have received a biodegradability certification. These suggestions encourage the transition to a circular economy by encouraging the use of reusable rather than single-use items, material recycling, and the use of biodegradable materials to lessen the environmental impact of waste disposal. In the years ahead, we plan to adapt the proposed PHFE unity research methods to other aspects, such as creating a class of algorithms based on PHFE evidence measures. The ranking results for the suggested WASPAS-MCDM techniques surpassed the existing PHFS-MCDM methods in the research study by providing the best solution. The validity of the proposed methods is evaluated by comparing them with other MCDM methods like COPRAS, and the obtained results show that the proposed methods are more efficient in handling uncertain data in the MCDM environment. The sensitivity analysis examines the ranking order when subjective weights’ importance is changed in two cases. Our solutions provide the most effective technique for reducing the negative effects of bio-plastic bags on the environment. The proposed method can be extended to interval-valued neutrosophic, dual hesitant fuzzy sets, and spherical fuzzy sets in future works.

## Data Availability

Data sharing not applicable to this article as no datasets were generated or analysed during the current study.
